# *circ-hnRNPU* inhibits NONO-mediated c-Myc transactivation and mRNA stabilization essential for glycosylation and cancer progression

**DOI:** 10.1186/s13046-023-02898-5

**Published:** 2023-11-23

**Authors:** Hongjun Li, Wanju Jiao, Jiyu Song, Jianqun Wang, Guo Chen, Dan Li, Xiaojing Wang, Banghe Bao, Xinyi Du, Yang Cheng, Chunhui Yang, Qiangsong Tong, Liduan Zheng

**Affiliations:** 1 Department of Pathology, Union Hospital, Tongji Medical College, Huazhong University of Science and Technology, 1277 Jiefang Avenue, Wuhan, Hubei Province 430022 P. R. China; 2 Department of Pediatric Surgery, Union Hospital, Tongji Medical College, Huazhong University of Science and Technology, 1277 Jiefang Avenue, Wuhan, Hubei Province 430022 P. R. China; 3 Department of Geriatrics, Union Hospital, Tongji Medical College, Huazhong University of Science and Technology, 1277 Jiefang Avenue, Wuhan, Hubei Province 430022 P. R. China

**Keywords:** Gastric cancer, Circular RNA, Heterogenous nuclear ribonuclear protein U, Non-POU domain containing octamer binding, Glycosylation

## Abstract

**Background:**

Recent evidence reveals the emerging functions of circular RNA (circRNA) and protein glycosylation in cancer progression. However, the roles of circRNA in regulating glycosyltransferase expression in gastric cancer remain to be determined.

**Methods:**

Circular RNAs (circRNAs) were validated by Sanger sequencing. Co-immunoprecipitation, mass spectrometry, and RNA sequencing assays were applied to explore protein interaction and target genes. Gene expression regulation was observed by chromatin immunoprecipitation, RNA immunoprecipitation, dual-luciferase reporter, real-time quantitative RT-PCR, and western blot assays. Gain- and loss-of-function studies were performed to observe the impacts of circRNA and its partners on the glycosylation, growth, invasion, and metastasis of gastric cancer cells.

**Results:**

*Circ-hnRNPU*, an exonic circRNA derived from heterogenous nuclear ribonuclear protein U (*hnRNPU*), was identified to exert tumor suppressive roles in protein glycosylation and progression of gastric cancer. Mechanistically, *circ-hnRNPU* physically interacted with non-POU domain containing octamer binding (NONO) protein to induce its cytoplasmic retention, resulting in down-regulation of glycosyltransferases (*GALNT2*, *GALNT6*, *MGAT1*) and parental gene *hnRNPU* via repression of nuclear NONO-mediated c-Myc transactivation or cytoplasmic NONO-facilitated mRNA stability. Rescue studies indicated that *circ-hnRNPU* inhibited the N- and O-glycosylation, growth, invasion, and metastasis of gastric cancer cells via interacting with NONO protein. Pre-clinically, administration of lentivirus carrying *circ-hnRNPU* suppressed the protein glycosylation, tumorigenesis, and aggressiveness of gastric cancer xenografts. In clinical cases, low *circ-hnRNPU* levels and high *NONO* or *c-Myc* expression were associated with poor survival outcome of gastric cancer patients.

**Conclusions:**

These findings indicate that *circ-hnRNPU* inhibits NONO-mediated c-Myc transactivation and mRNA stabilization essential for glycosylation and cancer progression.

**Supplementary Information:**

The online version contains supplementary material available at 10.1186/s13046-023-02898-5.

## Background

Gastric cancer is one of the leading causes of cancer-related death around the world [1]. In spite of improvement in surgery, chemotherapy, and radiotherapy, the outcome of gastric cancer patients at advanced stages still remains poor, mainly due to tumor recurrence, invasion, and metastasis [1]. Therefore, discovery of novel therapeutic approaches is urgent for improving prognosis of gastric cancer. As an important type of post-translational modification, glycosylation affects stabilization, localization, or activity of proteins, and contributes to cancer progression [2]. For example, O-linked beta-N-acetylglucosamine (O-GlcNAc) modification regulates the expression of c-Myc [3] and p21 [4] during epithelial-mesenchymal transition or cell cycle process, affects trafficking of epidermal growth factor receptor in signaling transduction [5], and facilitates activities of malate dehydrogenase 1 crucial for pancreatic cancer progression [6]. Similarly, N-linked glycosylation modulates the activity or signaling of cell surface proteins, such as insulin-like growth factor 1 receptor, fibroblast growth factor, and platelet-derived growth factor, and is associated with cancer metastasis [7]. However, the mechanisms regulating glycosyltransferase expression in gastric cancer remain to be determined.

Circular RNA (circRNA) is a type of endogenous single-stranded and covalently closed non-coding RNA [8], and classified into exonic, intronic, and exon–intron ones [9]. Certain circRNAs, such as *ciRS-7* and circular sex determining region Y RNA, serve as sponge of microRNAs to compete their inhibitory effects on gene expression [10]. In addition, circRNAs such as *circ-Foxo3* [11] and *circ-HuR* [12], acting as protein baits or antagonists, can affect the transport, storage, and functional effectiveness of RNA binding protein (RBP). Meanwhile, many circRNAs are also able to recruit ribosome and encode proteins or peptides via internal ribosome entry site- or N6-methyladenosine -mediated translation initiation [13, 14]. It has been established that circRNAs are aberrantly expressed in multiple types of cancer, such as gastric cancer, hepatocellular carcinoma, lung cancer, colon carcinoma, and leukaemia [8]. However, the regulatory roles and mechanisms of circRNAs in protein glycosylation and gastric cancer progression warrant investigation.

In this study, we identify a circRNA derived from exons 5, 6, 7 and 8 of heterogeneous nuclear ribonucleoprotein U (*hnRNPU*), an oncogene with multiple important functions [15], which is termed as *circ-hnRNPU* that acts as a tumor suppressor in glycosylation and progression of gastric cancer. Mechanistically, *circ-hnRNPU* directly interacts with and triggers cytoplasmic retention of non-POU domain containing octamer binding (NONO) protein, resulting in down-regulation of glycosyltransferases and parental gene *hnRNPU* via repression of nuclear NONO-mediated c-Myc transactivation and cytoplasmic NONO-facilitated mRNA stabilization. In addition, *circ-hnRNPU* inhibits glycosylation, growth, invasion, and metastasis of gastric cancer cells via interacting with NONO protein, providing a therapeutic target for protein glycosylation and cancer progression.

## Methods

### Cell culture

Human gastric cancer cells AGS (CRL-1739), MGC-803 (JCRB0254), MKN-45 (JCRB0254), NCI-N87 (CRL-5822), cervical cancer HeLa cells (CCL-2), prostate cancer PC-3 cells (CRL-1435), embryonic kidney HEK293T cells (CRL-11268), and normal gastric epithelial GES-1 cells (C6268) were obtained from Japanese Collection of Research Bioresources Cell Bank (Osaka, Japan), American Type Culture Collection (Rockville, MD), or Beyotime Biotechnology (Beijing, China). Cells were authenticated by short tandem repeat profiling, and used within 6 months after resuscitation of frozen aliquots. Mycoplasma contamination was regularly examined using Lookout Mycoplasma PCR Detection Kit (MP0035, Sigma, St. Louis, MO). Cell lines were cultured in RPMI 1640 medium supplied with 10% fetal bovine serum (Gibco, Carlsbad, CA) in a humidified atmosphere of 5% CO_2_ at 37 °C, and treated with actinomycin D (ActD, Sigma), benzyl-α-GalNAc (BAG, Sigma), or tunicamycin (Tu, Abcam Inc., Cambridge, MA) as indicated.

### RT-PCR and real-time quantitative RT-PCR (qRT-PCR)

Nuclear or cytoplasmic RNA was isolated with RNA Subcellular Isolation Kit (Active Motif, Carlsbad, CA). Genomic DNA (gDNA) and total RNA were extracted with DNA Mini Kit (Qiagen, Valencia, CA) and RNeasy Mini Kit (Qiagen), respectively. For circRNA detection, RNase R digestion (3 U/mg, Epicenter, Madison, WI) was performed at 37 °C for 15 min. Complementary DNA was synthesized with Transcriptor First Strand cDNA Synthesis Kit (Roche, Indianapolis, IN). Quantification of mRNA, circRNA, or gDNA was undertaken with SYBR Green PCR Master Mix (Applied Biosystems, Foster City, CA) and primers (Additional file 1: Table S1). To measure mRNA stability, de novo RNA synthesis was blocked with ActD (5 μg/ml), while half-life of mRNA was examined by comparing transcript levels before and after ActD treatment.

### Northern blot

The junction-specific probes for *circ-hnRNPU* (Additional file 1: Table S2) were synthesized and labeled by digoxin (DIG) at TSINGKE (Wuhan, China). Northern blotting was conducted as previously reported [16], and recorded on X-ray films with chemiluminescence substrate CSPD (Roche).

### RNA fluorescence in situ hybridization (RNA-FISH)

Biotin-labeled antisense or sense oligonucleotide probe targeting *circ-hnRNPU* junction (Additional file 1: Table S2) was synthesized by TSINGKE, while probes for *GAPDH* and *U1* were synthesized by in vitro transcription of PCR fragments (Additional file 1: Table S1) with biotin RNA Labeling Mix and T7 RNA polymerase (Roche). Hybridization was undertaken in a humidified chamber at 37 °C for 16 h with or without RNase R (3 U/mg) treatment. The signals were detected by using Fluorescent In Situ Hybridization kit (RiboBio, Guangzhou, China), while nuclei were counterstained with 4′,6-diamidino-2-phenylindole (DAPI, Sigma).

### Western blot

Tissue or cellular protein was extracted by using 1 × cell lysis buffer (Promega, Madison, WI). Cytoplasmic or nuclear fractions were isolated using NE-PER Nuclear and Cytoplasmic Extraction Reagents (Thermo Fisher Scientific, Inc., Grand Island, NY). Western blot was performed as previously described [17, 18], with antibodies specific for O-GlcNAc (#12,938), maltose binding protein (MBP, #2396, Cell Signaling Technology, Danvers, MA), hnRNPU (ab10297), NONO (ab70335), c-Myc (ab32072), Flag-tag (ab45766), hemagglutinin (HA)-tag (ab9110), glutathione S-transferase (GST)-tag (ab36415), polypeptide N-acetylgalactosaminyl-transferase 6 (GALNT6, ab151329), fibronectin 1 (FN1, sc-59826, Santa Cruz Biotechnology, Santa Cruz, CA), alpha-1,3-mannosyl-glycoprotein 2-beta-N- acetylglucosaminyltransferase (MGAT1, ab180578), N-glycosylated protein glucose transporter 1 (GLUT1, ab115730), polypeptide N-acetylgalacto-saminyltransferase 2 (GALNT2, ab140637), glyceraldehyde-3-phosphate dehydrogenase (GAPDH, ab8245), histone H3 (ab1791), or β-actin (ab6276, Abcam Inc.).

### Lectin affinity precipitation

Cell lysates containing 600 μg of protein were incubated with N-glycans-recognizing biotinylated phaseolus vulgaris lectin (PHA-E + L, 21510096–1, Glycomatrix, Dublin, OH) conjugated to agarose beads overnight at 4 °C, then with 20 μl of streptavidin-conjugated agarose (Thermo Fisher Scientific, Inc.) for 2 h. Glycoprotein/lectin complexes were collected by brief centrifugation (1400 rpm, 5 min, 4 °C), separated on a 12% denaturing sodium dodecyl sulfate (SDS) gel, and subjected to western blotting assay.

### Gene over-expression or knockdown

The *circ-hnRNPU* (594 bp) with circularization framework sites (Additional file 1: Table S3) was synthesized by TSINGKE and subcloned into pLCDH-ciR (Geenseed Biotech Co., Guangzhou, China). To generate corresponding construct of linear *circ-hnRNPU* (lin*-hnRNPU*), the reverse circularization framework sites were removed by QuikChange II Site-Directed Mutagenesis Kit (Agilent, Santa Clara, CA) and primers (Additional file 1: Table S3). Human *NONO* cDNA (1416 bp) and *c-Myc* cDNA (1365 bp) were provided by Drs. Jean-Yves Masson [19] and William P. Tansey [20], respectively. Their truncations were amplified with PCR primers (Additional file 1: Table S3) and subcloned into pCMV-3Tag-1A, pCMV-HA, pMAL-c4X, or pGEX-6P-1 (Addgene, Cambridge, MA), respectively. Mutation of nuclear localization signal (NLS) was undertaken with QuikChange II Site-Directed Mutagenesis Kit (Agilent) and primer sets (Additional file 1: Table S3). Human *hnRNPU*, *GALNT6* and *MGAT1* vectors were obtained from GeneChem Co., Ltd (Shanghai, China). Oligonucleotides specific for short hairpin RNAs (shRNAs) targeting *circ-hnRNPU*, *NONO*, or *c-Myc* (Additional file 1: Table S2) were synthesized by TSINGKE and inserted into GV298 (GeneChem Co., Ltd). Lentiviral vectors were co-transfected with packaging plasmids psPAX2 and pMD2G (Addgene) into HEK293T cells. Stable cell lines were obtained by selection for neomycin or puromycin (Invitrogen, Carlsbad, CA) resistance.

### Rescue of target gene expression

To rescue *circ-hnRNPU* over-expression-altered gene expression, *NONO* or *c-Myc* construct was transfected into stable cell lines. To restore gene expression induced by *circ-hnRNPU* silencing, shRNAs against *NONO* or *c-Myc* (Additional file 1: Table S2) were transfected into cancer cells with Genesilencer Transfection Reagent (Genlantis, San Diego, CA).

### RNA sequencing (RNA-seq)

Total RNA of cancer cells (1 × 10^6^) was extracted with TRIzol® reagent (Life Technologies, Inc.). Library preparation and transcriptome sequencing on an Illumina HiSeq X Ten platform were carried out at Wuhan SeqHealth Technology Co., Ltd. (Wuhan, China). Gene set enrichment analysis (GSEA) was undertaken as previously reported [17], with application of indicated gene sets.

### Biotin-labeled RNA pull-down and mass spectrometry

Biotin-labeled oligonucleotide probes targeting junction sites of circRNAs were synthesized (Invitrogen, Additional file 1: Table S2). The lysates of 2 × 10^7^ cancer cells were incubated with 3 μg of biotin-labeled probe for 2 h, and incubated with 35 μl of Streptavidin C1 magnetic beads (Invitrogen) for 1 h. Retrieved proteins were detected through western blot or mass spectrometry at SpecAlly Life Technology Co., Ltd (Wuhan, China).

### Cross-linking RNA immunoprecipitation (RIP)

Cells were cross-linked by ultraviolet light (200 J/cm^2^, 254 nm). RIP assay was undertaken with Magna RIP™ RNA-Binding Protein Immunoprecipitation Kit (Millipore, Temecula, CA), using antibodies specific for NONO (ab70335) or Flag-tag (ab45766, Abcam Inc.) and protein A-Sepharose beads (Santa Cruz Biotechnology) at 4 °C for 4 h. Co-precipitated RNAs were detected by RT-PCR or real-time qRT-PCR with primers (Additional file 1: Table S1).

### RNA electrophoretic mobility shift assay (EMSA)

The 5'-monophosphorylated linear *circ-hnRNPU* was in vitro transcribed by using Biotin RNA Labeling Mix (Roche) and T7 RNA polymerase, and circularized with guide oligonucleotide targeting circular junction (Additional file 1: Table S2) and T4 RNA ligase I (Qiagen) [21]. RNA EMSA assay using recombinant NONO protein and biotin-labeled circular probe of *circ-hnRNPU* was performed with LightShift Chemiluminescent RNA EMSA Kit (Thermo Fisher Scientific, Inc.).

### In vitro binding assay

By using the FLAG® M Purification Kit (Sigma), Flag-tagged NONO protein was prepared from HEK293 cells transfected by corresponding constructs. The GST-tagged NONO proteins were produced from *E. coli* as previously described [21]. The Flag-tagged NONO protein was purified by using FLAG® M Purification Kit (Millipore). The GST- or Flag-tagged NONO protein and biotin-labeled circular probe of *circ-hnRNPU* were pulled down by using Flag or GST beads (Sigma). *Circ-hnRNPU* was validated by RT-PCR using divergent primers (Additional file 1: Table S1), while protein was detected by western blot.

### Co-immunoprecipitation (co-IP) assay

Co-IP was performed as previously described [17, 18, 21], with antibodies (1:200 dilutions) specific for NONO (ab70335), c-Myc (ab32072), Flag-tag (ab45766), HA-tag (ab9110), GST-tag (ab36415, Abcam Inc.), or MBP (#2396, Cell Signaling Technology). Bead-bound proteins were released and analyzed by western blot.

### Fluorescence immunocytochemical staining

Cancer cells were grown on coverslips, incubated with antibody specific for NONO (ab70335, Abcam lnc.; 1:100 dilution) or c-Myc (ab32072, Abcam lnc.; 1:100 dilution) at 4 °C overnight, and treated with fluorescein isothiocyanate-conjugated goat anti-rabbit IgG (1:1000 dilution) and DAPI (300 nM) staining.

### Bimolecular fluorescence complementation (BiFC) assay

Human *NONO* cDNA (1416 bp) and *c-Myc* cDNA (1365 bp) were respectively subcloned into BiFC vectors pBiFC-VN173 or pBiFC-VC155 (Addgene), and co-transfected into cancer cells for 24 h. The fluorescence emission was observed under a confocal microscope, with excitation and emission wavelengths of 488 and 500 nm, respectively [17, 18].

### Chromatin immunoprecipitation (ChIP) assay

ChIP assay was undertaken by using EZ-ChIP kit (Upstate Biotechnology, Temacula, CA), with antibodies (1:100 dilution) specific for c-Myc (#18,583, Cell Signaling Technology, Inc.) and primers targeting gene promoters (Additional file 1: Table S1).

### Dual-luciferase reporter assay

Complementary oligonucleotides containing three or four canonical binding sites of transcription factors, and promoter fragments of *GALNT6* (-460/ + 50) or *MGAT1* (-1595/-1196) amplified from genomic DNA (Additional file 1: Table S3) were subcloned into pGL3-Basic (Promega). Human *NONO* activity reporter was established by inserting complementary oligonucleotides containing four canonicial NONO binding sites (Additional file 1: Table S3) into 3'-UTR of *Renilla* luciferase within psiCHECK2 (Promega). Dual-luciferase assay was performed as previously reported [18, 22].

### In vitro cell viability, growth, migration, and invasion assays

The 2-(4,5-dimethyltriazol-2-yl)-2,5-diphenyl tetrazolium bromide (MTT, Sigma) colorimetric, soft agar, and matrigel invasion assays were conducted to measure the viability, growth, and invasive capabilities of cancer cells in vitro [17, 21, 22].

### In vivo growth, metastasis, and therapeutic assays

All animal experiments were approved by the Experimental Animal Ethics, Huazhong University of Science and Technology, and undertaken according to NIH Guidelines for the Care and Use of Laboratory Animals. For in vivo tumor growth and experimental metastasis studies, cancer cells (1 × 10^6^ or 0.4 × 10^6^) were injected into dorsal flanks or tail vein of blindly randomized four-week-old male BALB/c nude mice (National Rodent Seeds Center, Shanghai, China) [17, 18, 21]. For in vivo therapeutic studies, cancer cells (1 × 10^6^ or 0.4 × 10^6^) were injected into dorsal flanks or tail vein of nude mice, respectively. One week later, mice were blindly randomized and treated by tail vein injection of lentivirus (1 × 10^7^ plaque-forming units) as indicated. The in vivo Optical Imaging System (In-Vivo FX PRO, Bruker Corporation, Billerica, MA) was applied to acquire fluorescent images of xenograft tumors in nude mice.

### Patient tissue samples

Human tissue study was approved by the Institutional Review Board of Union Hospital, Tongji Medical College. All procedures were conformed to principles set forth by Declaration of Helsinki. Written informed consent was obtained from all patients without preoperative chemotherapy or other treatment. Fresh cancer tissues were collected at surgery, validated by pathological diagnosis, and stored at -80 °C.

### Immunohistochemistry

Immunohistochemical staining and quantitative evaluation were performed as previously described [17, 18], with antibodies specific for Ki-67 (1:500, sc-23900, Santa Cruz Biotechnology), CD31 (1:500, ARG52748, Arigo, Hsinchu City, Taiwan), or NONO (1:500, ab70335, Abcam Inc.).

### Statistical analysis

All data were shown as mean ± standard error of the mean (s.e.m.). Cutoff values for gene expression were defined by medium or average levels. Student’s *t* test, analysis of variance (ANOVA), Mann–Whitney U test, and χ^2^ analysis were used to evaluate differences. Statistical significance of overlap between two gene lists was determined by Fisher’s exact test. Pearson’s correlation coefficient assay was used to analyze expression correlation. Log-rank test was used to assess survival difference. All statistical tests were two-sided and considered statistically significant when *P* values were less than 0.05.

## Results

### Low *circ-hnRNPU* expression is associated with poor prognosis of gastric cancer

**Fig. 1:**
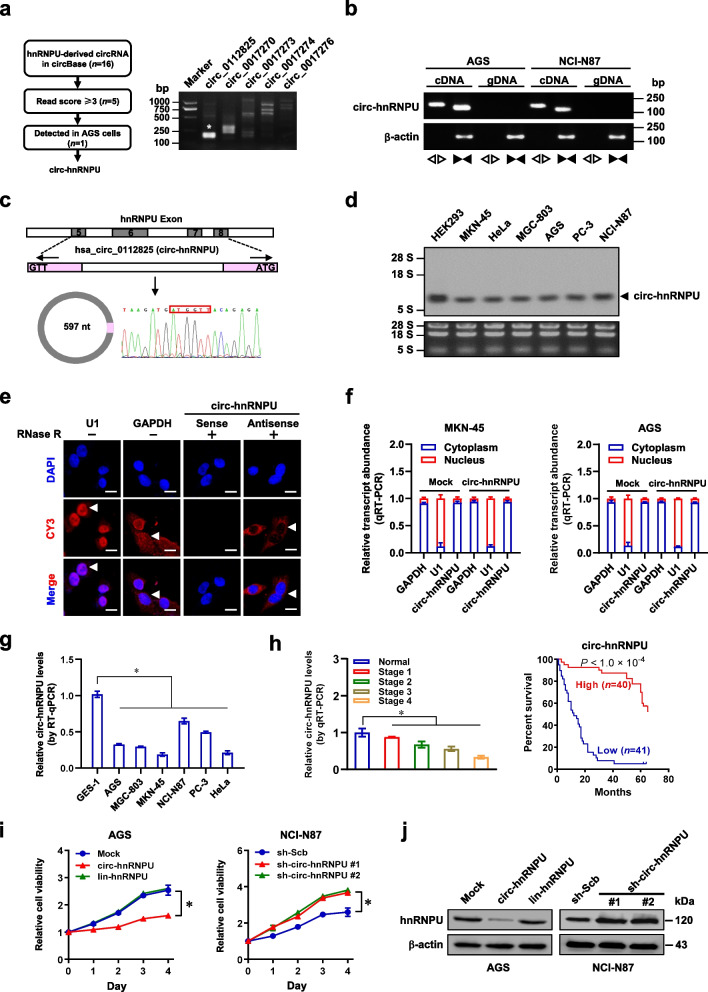
Low *circ-hnRNPU* expression is associated with poor prognosis of gastric cancer. **a**, Flowchart (left panel) and RT-PCR assay with divergent primers (right panel) indicating the identification of detectable circRNA (asterisk) generated from *hnRNPU* in gastric cancer AGS cells. **b**, PCR assay indicating the amplification of *circ-hnRNPU* with divergent or convergent primers from cDNA or gDNA of gastric cancer cell lines AGS and NCI-N87, while *β-actin* was used as a negative control. **c**, Schematic illustration showing the genomic location and composition of *circ-hnRNPU* generated from its host gene, and validation by Sanger sequencing. **d**, Northern blot assay using a junction-specific antisense probe indicating the existence of 597-nt *circ-hnRNPU* in indicated cell lines. **e**, RNA-FISH using CY3-labeled antisense junction probe showing the localization of *circ-hnRNPU* (red, arrowheads) in NCI-N87 cells, with or without RNase R digestion (3 U/mg), while sense probe, *GAPDH*, or *U1* were applied as negative or positive controls. Scale bar: 10 μm. **f**, Real-time qRT-PCR (normalized to *β-actin*) indicating the distribution of *GAPDH*, *U1*, and *circ-hnRNPU* in the cytoplasmic and nuclear fractions of MKN-45 and AGS cells stably transfected with empty vector (mock) or *circ-hnRNPU* (*n* = 5). **g**, Real-time qRT-PCR assay indicating the levels of *circ-hnRNPU* (normalized to *β-actin*, *n* = 4) in cultured GES-1 and cancer cells. **h**, Real-time qRT-PCR (left panel, normalized to *β-actin*) assay indicating the *circ-hnRNPU* levels in normal gastric mucosa (*n* = 40) and gastric cancer tissues (*n* = 81). Kaplan–Meier curves (right panel) indicating the survival of gastric cancer patients (*n* = 81) with low or high expression of *circ-hnRNPU* (cutoff value = 0.58). **i**, MTT colorimetric assay showing the viability of AGS and NCI-N87 cells stably transfected with mock, *circ-hnRNPU*, linear *circ-hnRNPU* (*lin-hnRNPU*), scramble shRNA (sh-Scb), sh-*circ-hnRNPU* #1, or sh-*circ-hnRNPU* #2. **j**, Western blot assay revealing the hnRNPU protein levels in AGS and NCI-N87 cells stably transfected with mock, *circ-hnRNPU*, *lin-hnRNPU*, sh-Scb, sh-*circ-hnRNPU* #1, or sh-*circ-hnRNPU* #2. Student’s *t* test or analysis of variance compared the difference in **g-i**. *P < 0.05. Data are shown as mean ± s.e.m. (error bars) or representative of three independent experiments in **a**, **b**, **d**-**g**, **i** and **j**

To investigate the roles of circular transcript derived from oncogenic *hnRNPU*, 16 potential circRNAs were discovered through mining of circBase database (http://www.circbase.org), with 5 circular transcripts having more than 3 read scores (Fig. 1a). Among them, only *hsa_circ_0112825* (termed as *circ-hnRNPU*) was detectable in gastric cancer AGS cells (Fig. 1a), which was further validated by amplification using convergent or divergent primers (Fig. 1b). The accurate cyclization of *circ-hnRNPU*, consisting of exons 5, 6, 7 and 8 of *hnRNPU*, was confirmed by Sanger sequencing (Fig. 1c). Northern blot assay using a junction-specific probe indicated the existence of 597-nt *circ-hnRNPU* in a variety of cancer cell lines (Fig. 1d), and those stably transfected with empty vector (mock) or *circ-hnRNPU* (Additional file 1: Fig. S1a). The RNase R resistant characteristics were further observed in endogenous and exogenous *circ-hnRNPU* (Additional file 1: Fig. S1a, b), which was mainly localized at cytoplasm of gastric cancer cells (Fig. 1e, f). There was decreased *circ-hnRNPU* expression in gastric cancer cell lines (AGS, MGC-803, MKN-45, and NCI-N87), prostate cancer PC-3 cells, and cervical cancer HeLa cells, when compared to that of GES-1 cells (Fig. 1g). Lower *circ-hnRNPU* levels were observed in gastric cancer specimens, when compared with normal gastric mucosa, especially in those of advanced clinical stages (Fig. 1h), which were associated with poor survival probability of patients (*P* < 1.0 × 10^–4^, Fig. 1h). Based on lower and upper quartiles, the *circ-hnRNPU* levels in cancer cells were classified into relatively low, medium and high abundance. Among them, MKN-45, HeLa (relatively low), AGS (relatively middle), NCI-N87, and PC-3 (relatively high) were chosen as models for investigating functions of *circ-hnRNPU*. Stable transfection of *circ-hnRNPU*, but not of linear construct with removal of circularization framework sites (lin-*hnRNPU*), resulted in increase of *circ-hnRNPU* levels in MKN-45, AGS and HeLa cells (Additional file 1: Fig. S1c). Meanwhile, reduced *circ-hnRNPU* expression was observed in NCI-N87, AGS, or PC-3 cells stably transfected with shRNAs targeting junction site of *circ-hnRNPU* (Additional file 1: Fig. S1c). Notably, stable over-expression or knockdown of *circ-hnRNPU* reduced or facilitated the viability of AGS and NCI-N87 cells (Fig. 1i), along with down-regulation or up-regulation of parental gene *hnRNPU* (Fig. 1j), respectively. However, ectopic expression of lin-*hnRNPU* did not affect the viability or *hnRNPU* levels of these cells (Fig. 1i, j). Consistently, over-expression of *circ-hnRNPU* inhibited the anchorage-independent growth and invasiveness of AGS cells, which were partly rescued by *hnRNPU* transfection (Additional file 1: Fig. S1d, e). These findings suggested that low *circ-hnRNPU* expression was associated with poor prognosis of gastric cancer.

### *Circ-hnRNPU* inhibits O- and N-glycosylation essential for aggressiveness of gastric cancer cells

**Fig. 2:**
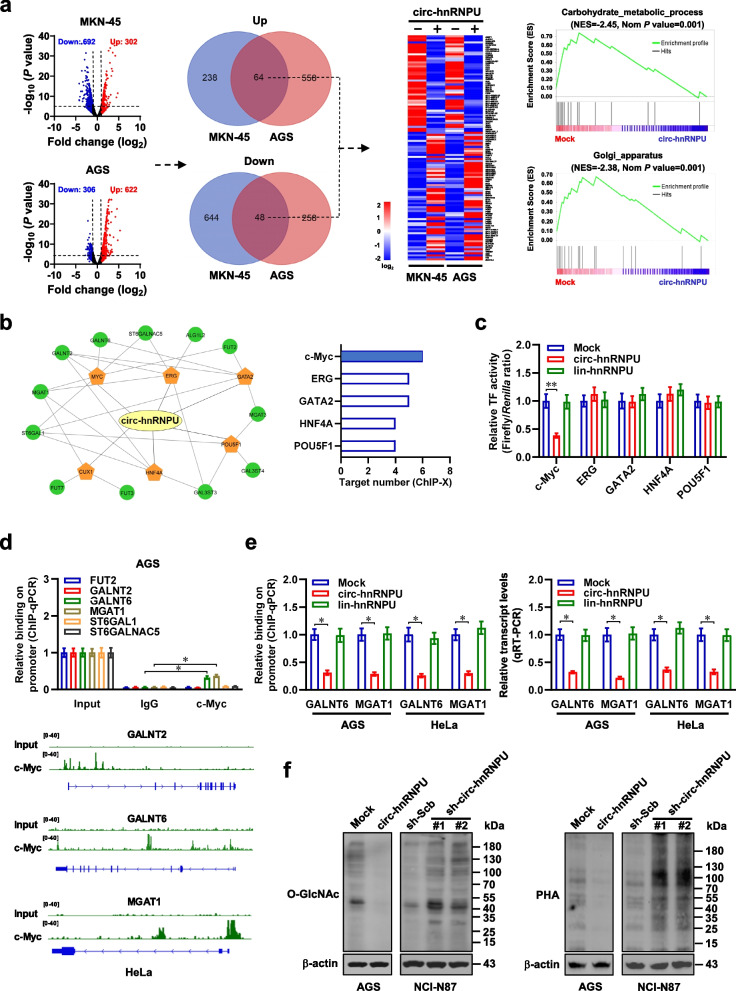
*Circ-hnRNPU* inhibits O- and N-glycosylation in gastric cancer cells. **a**, Volcano plots (left panel), Venn diagram (middle panel), heatmap (middle panel), and GSEA (right panel) showing the altered gene expression and corresponding biological processes in MKN-45 and AGS cells stably transfected with empty vector (mock) or *circ-hnRNPU*. **b**, Regulatory network (left panel) and ChIP-X analysis (right panel) showing the potential transcription factors for altered glycosyltransferase genes. **c**, Dual-luciferase assay revealing the activity of potential transcription factors in AGS cells stably transfected with mock or *circ-hnRNPU* (*n* = 5). **d**, ChIP and qPCR (upper panel) and ChIP-seq peak (GSE51334, lower panel) indicating endogenous enrichment of c-Myc on promoter regions of glycosyltransferases in AGS and HeLa cells. **e**, ChIP and qPCR (left panel) and real-time qRT-PCR (right panel, normalized to *β-actin*) assay showing the c-Myc enrichment and transcriptional levels of *GALNT6* and *MGAT1* in AGS and HeLa cells stably transfected with mock or *circ-hnRNPU* (*n* = 4)*.* **f**, Western blot assay indicating the levels of O- and N-glycosylation in AGS and NCI-N87 cells stably transfected with mock, *circ-hnRNPU*, scramble shRNA (sh-Scb), or sh-circ-hnRNPU. Student’s *t* test compared the difference in **c**-**e**. *P < 0.05, **P < 0.01. Data are shown as mean ± s.e.m. (error bars) or representative of three independent experiments in **c**-**f**

To further investigate downstream targets of *circ-hnRNPU*, RNA-seq assay was performed and indicated 64 consistently up-regulated and 48 down-regulated genes (fold change > 2.0, *P* < 0.05) in both MKN-45 and AGS cells upon *circ-hnRNPU* over-expression (Fig. 2a). GSEA assay revealed Golgi apparatus-associated carbohydrate metabolism as their top involved biological processes, while most of them were glycosyltransferases essential for protein glycosylation (Fig. 2a, Additional file 1: Fig. S1f). Further analysis by ChIP-X program [23] indicated potential transcription factors regulating these glycosyltransferase genes (Fig. 2b). Notably, among top five transcription factors ranking by target gene number, the activity of c-Myc, but not of ETS-related gene (ERG), GATA binding protein 2 (GATA2), hepatocyte nuclear factor 4 alpha (HNF4A), or POU class 5 homeobox 1 (POU5F1), was reduced by *circ-hnRNPU* over-expression in cultured AGS cells (Fig. 2c). Further ChIP and quantitative PCR (qPCR) assays revealing endogenous c-Myc enrichment on promoter regions of *GALNT6* and *MGAT1*, but not of other potential targets fucosyltransferase 2 (*FUT*2), *GALNT2*, ST6 beta-galactoside alpha-2,6-sialyltransferase 1 (*ST6GAL1*), or ST6 N-acetylgalactosaminide alpha-2,6-sialyltransferase 5 (*ST6GALNAC5*, Fig. 2d), which was attenuated by ectopic expression of *circ-hnRNPU*, but not of lin-*hnRNPU*, in AGS and HeLa cells (Fig. 2e). Accordingly, stable transfection of *circ-hnRNPU* led to reduction of *GALNT6* and *MGAT1* levels in these cells (Fig. 2e). Importantly, stable ectopic expression or silencing of *circ-hnRNPU* led to significant reduction or elevation of O- and N-glycosylation in gastric cancer AGS and NCI-N87 cells (Fig. 2f). Since protein glycosylation contributes to tumorigenesis and aggressiveness [24], we further investigated the influence of *circ-hnRNPU* on biological behaviors of gastric cancer cells. Knockdown of *circ-hnRNPU* increased the anchorage-independent growth and invasion capabilities of AGS cells, which was partially prevented by treatment with BAG or Tu, established inhibitors of O-glycosylation or N-glycosylation [25, 26] (Additional file 1: Fig. S2a, b). These results demonstrated that *circ-hnRNPU* inhibited O- and N-glycosylation essential for aggressiveness of gastric cancer cells.

### *Circ-hnRNPU* induces cytoplasmic retention of NONO via physical interaction

**Fig. 3:**
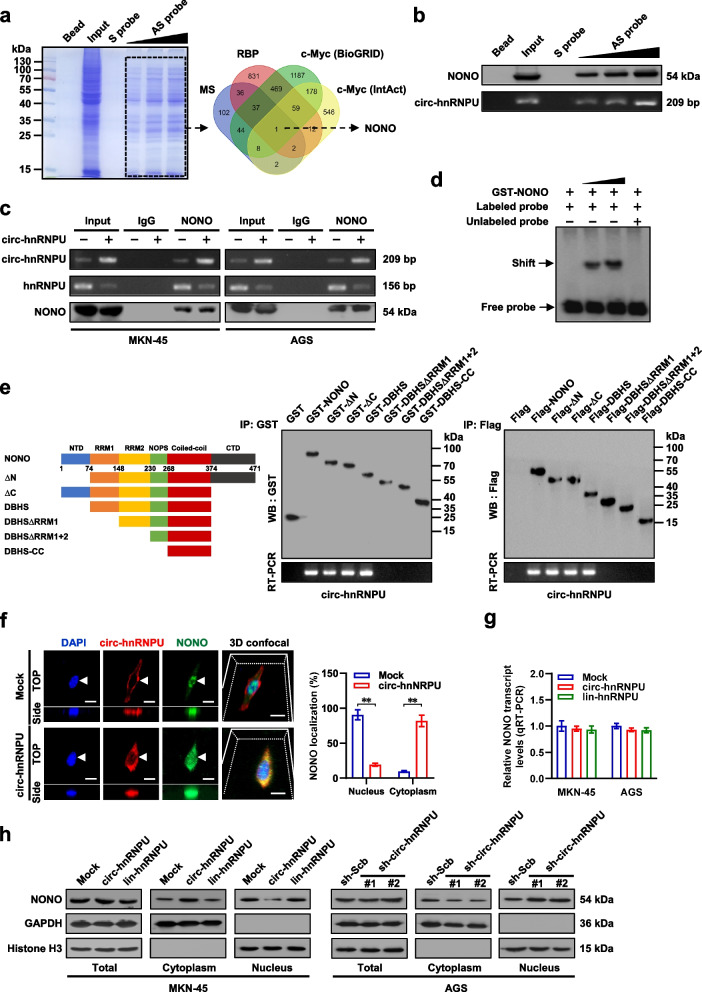
*Circ-hnRNPU* interacts with and induces cytoplasmic retention of NONO in gastric cancer cells. **a**, Coomassie bright blue staining (left panel) and Venn diagram (right panel) showing the differential proteins pulled down by biotin-labeled sense (S) or antisense (AS) probe targeting junction sites of *circ-hnRNPU* from the lysates of AGS cells, and overlapping analysis of proteins identified by mass spectrometry (MS) with established RBP from RBPDB (http://rbpdb.ccbr.utoronto.ca) and c-Myc-binding proteins derived from BioGRID and IntAct (https://www.ebi.ac.uk/intact) databases. **b**, Western blot (upper panel) and RT-PCR (lower panel) assays indicating the NONO protein or *circ-hnRNPU* pulled down by biotin-labeled S or AS probes targeting junction site of endogenous *circ-hnRNPU* from lysates of AGS cells. **c**, RIP (upper panel) and western blot (lower panel) assays using NONO antibody showing the interaction of NONO with *circ-hnRNPU* or *hnRNPU* mRNA in MKN-45 and AGS cells stably transfected with empty vector (mock) or *circ-hnRNPU*. **d**, RNA EMSA determining the interaction between recombinant GST-tagged NONO protein and biotin-labeled circular probe of *circ-hnRNPU*, with or without competition using an excess of unlabeled circular probe. **e**, Schematic diagram of *NONO* truncations (left panel) and in vitro binding assay (middle and right panels) depicting the recovered *circ-hnRNPU* levels detected by RT-PCR after incubation with full-length or truncation forms of GST-tagged or Flag-tagged recombinant *NONO* protein validated by western blot. **f**–**h**, Representative images and quantification of dual RNA-FISH and immunofluorescence (**f**), real-time qRT-PCR (**g**, normalized to *β-actin*), and western blot (**h**) assays indicated the localization of *circ-hnRNPU* and NONO (arrowheads), transcript and protein levels of *NONO* in MKN-45 and AGS cells stably transfected with mock, *circ-hnRNPU*, *lin-hnRNPU*, scramble shRNA (sh-Scb), or sh-*circ-hnRNPU*. Scale bar: 10 μm. Student’s *t* test compared the difference in **f** and **g**. **P < 0.01. Data are representative of three independent experiments in **b**-**h**

To explore potential protein partners of *circ-hnRNPU*, biotin-labeled RNA pull-down using probe targeting junction site was performed in AGS cells, followed by a proteomic analysis of RNA-associated protein complex (Fig. 3a). Mass spectrometry assay revealed 232 proteins immunoprecipitated by *circ-hnRNPU* antisense probe, but not by sense probe (Additional file 1: Table S4), while over-lapping analysis with c-Myc-binding proteins derived from BioGRID [27] and IntAct (https://www.ebi.ac.uk/intact) databases revealed NONO as the only interacting protein (Fig. 3a). Validating RNA pull-down assay indicated that *circ-hnRNPU* bound to NONO in the lysates of AGS cells in a dose-dependent manner (Fig. 3b). Cross-linking RIP assay also indicated the abundance of *circ-hnRNPU* within NONO antibody-immunoprecipitated RNA in a variety of cancer cell lines (Additional file 1: Fig. S2c, d), while steady transfection of *circ-hnRNPU* facilitated its interaction with NONO, resulting in decreased binding of NONO to *hnRNPU* mRNA (Fig. 3c). By using in vitro biotin-labeled cyclized probe, RNA EMSA indicated the direct interaction between *circ-hnRNPU* and recombinant GST-tagged NONO protein (Fig. 3d). To investigate their interaction domains, GST-tagged or Flag-tagged NONO truncation proteins were established (Fig. 3e). In vitro binding assays confirmed that RNA recognition motif 1 [RRM1, 74–148 amino acids (aa)] of NONO protein, rather than other domains, was essential for its interaction with *circ-hnRNPU* (Fig. 3e). Notably, dual RNA-FISH and immunofluorescence, real-time qRT-PCR, and western blot assays indicated that stable over-expression or knockdown of *circ-hnRNPU*, but not of lin*-hnRNPU*, led to obvious cytoplasmic retention or absence of NONO in gastric cancer cells, without alteration in *NONO* transcript or protein levels (Fig. 3f-h, Additional file 1: Fig. S2e). These results suggested that *circ-hnRNPU* induced cytoplasmic retention of NONO via physical interaction in gastric cancer.

### *Circ-hnRNPU* inhibits the interaction of NONO with c-Myc in gastric cancer cells

**Fig. 4:**
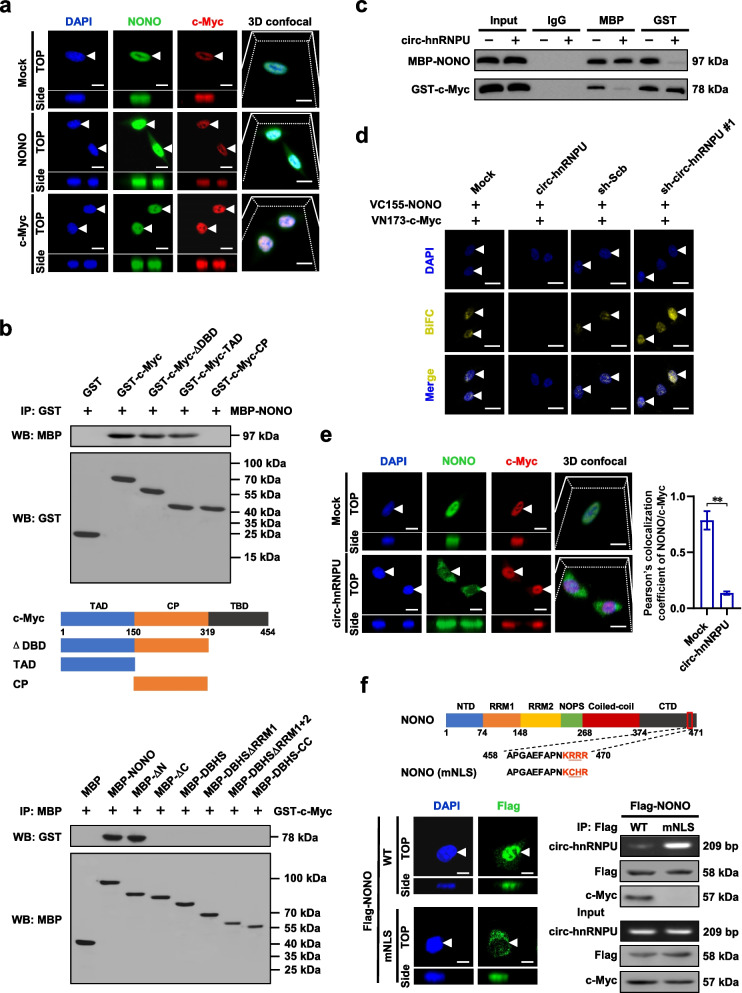
*Circ-hnRNPU* inhibits interaction of NONO with c-Myc in gastric cancer cells. a, Immunofluorescence assay showing the localization of NONO and c-Myc protein (arrowheads) in AGS cells stably transfected with empty vector (mock), *NONO*, or *c-Myc*. Scale bar: 10 μm. **b**, Schematic diagram, co-IP and western blot assays indicating the interaction between full-length or truncations of recombinant GST-tagged c-Myc and MBP-tagged NONO proteins. **c**, Co-IP and western blot assays revealing the direct interaction between recombinant MBP-tagged NONO and GST-tagged c-Myc proteins, with or without treatment by in vitro generated *circ-hnRNPU*. **d**, BiFC assay showing the interaction between NONO and c-Myc (arrowheads) in AGS cells stably transfected with mock, *circ-hnRNPU*, scramble shRNA (sh-Scb), or sh-circ-hnRNPU #1. Scale bar: 10 μm. **e**, Representative images (left panel) and quantification (right panel) of immunofluorescence assay revealing the co-localization of NONO and c-Myc (arrowheads) in AGS cells stably transfected with mock or *circ-hnRNPU*. Scale bar: 10 μm. **f**, Immunofluorescence (lower left panel), RIP and western blot (lower right panel) assays showing the localization (arrowheads) and interaction of NONO with *circ-hnRNPU* or c-Myc protein in AGS cells transfected with Flag-tagged wild-type (WT) or NLS-mutant (mNLS) form of *NONO* as indicated (upper panel). Student’s *t* test compared the difference in **e**. **P < 0.01. Data are representative of three independent experiments in **a**-**f**

Since above evidence revealing c-Myc as a potential transcription factor for glycosyltransferase expression, we hypothesized that *circ-hnRNPU* might affect the interaction of NONO with c-Myc. In cultured gastric cancer AGS cells, nuclear NONO was co-localized with c-Myc protein, while ectopic expression of *NONO* did not affect the nuclear localization of c-Myc (Fig. 4a). By using purified recombinant GST-tagged c-Myc and MBP-tagged NONO proteins, co-IP and western blot assays indicated their direct interaction (Fig. 4b). In addition, the transactivating domain (TAD, 1–150 aa) of c-Myc was required for its binding to NONO, while carboxyl-terminal domain (CTD, 375–471 aa) of NONO was crucial for its interaction with c-Myc (Fig. 4b, Additional file 1: Fig. S3a), which was further validated by results from AGS cells transfected with HA-tagged and Flag-tagged constructs (Additional file 1: Fig. S3b). Notably, incubation with cyclized product of *circ-hnRNPU* attenuated the direct interaction between MBP-tagged NONO and GST-tagged c-Myc proteins in vitro (Fig. 4c). BiFC assay further revealed the interaction between NONO and c-Myc in AGS cells, which was decreased or increased by stably ectopic expression or silencing of *circ-hnRNPU*, respectively (Fig. 4d). Consistently, ectopic expression of *circ-hnRNPU* significantly reduced the nuclear co-localization of NONO and c-Myc in AGS cells (Fig. 4e). Mutation of NLS analyzed by NLStradamus program [28] abolished the nuclear localization of NONO protein, which facilitated its interaction with *circ-hnRNPU* rather than c-Myc in AGS cells (Fig. 4f). These data indicated that *circ-hnRNPU* inhibited the interaction of NONO with c-Myc in gastric cancer cells.

### *Circ-hnRNPU* inhibits dual NONO activity in regulating c-Myc transactivation and mRNA stabilization

**Fig. 5:**
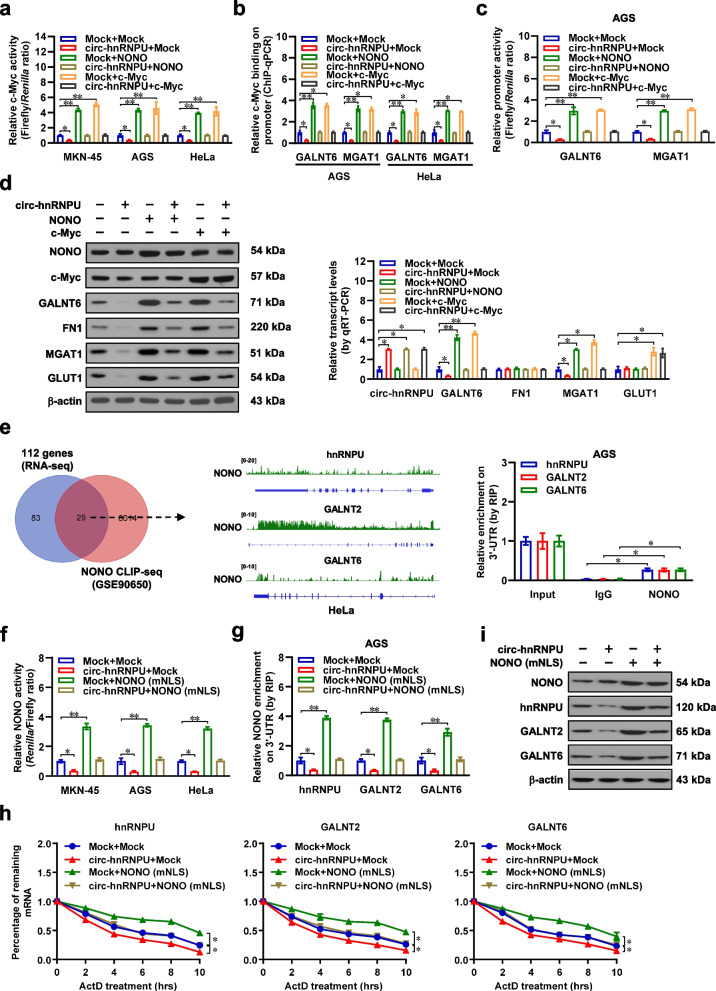
*Circ-hnRNPU* inhibits dual NONO activity in regulating c-Myc transactivation and mRNA stabilization. **a** and **b**, Dual-luciferase assay using a reporter containing three canonical c-Myc binding sites (**a**), ChIP and qPCR (b, normalized to input) assays indicating c-Myc transactivation and enrichment on target gene promoters in MKN-45, AGS, and HeLa cells stably transfected with empty vector (mock) or *circ-hnRNPU*, and those co-transfected with *NONO* or *c-Myc* (*n* = 5). **c** and **d**, Dual-luciferase assay (**c**), western blot (d, left panel), and real-time qRT-PCR (d, normalized to *β-actin*, right panel) assays showing promoter activity or expression of *GALNT6* and *MGAT1*, as well as *circ-hnRNPU* or downstream targets (*FN1* and *GLUT1*) levels in AGS cells stably transfected with mock or *circ-hnRNPU*, and those co-transfected with *NONO* or *c-Myc*. **e**, Venn diagram (left pane) and CLIP-seq peak (middle panel) indicating comprehensive analysis of 112 altered genes in RNA-seq and NONO-binding targets (GSE90650). RIP and real-time qRT-PCR assays (right panel) revealing endogenous NONO binding to 3'-UTR of *hnRNPU*, *GALNT2*, and *GALNT6* in AGS cells. **f** and **g**, Dual-luciferase assay using a 3'-UTR reporter containing four canonical NONO binding sites (f), RIP and real-time qRT-PCR (g) assays showing the activity and enrichment of NONO on 3'-UTR of *hnRNPU*, *GALNT2*, and *GALNT6* in MKN-45, AGS, and HeLa cells stably transfected with mock or *circ-hnRNPU*, and those co-transfected with NLS-mutant (mNLS) form of *NONO* (*n* = 5)*.* **h**, Real-time qRT-PCR (normalized to *β-actin*, *n* = 5) assays showing the mRNA stability of *hnRNPU*, *GALNT2*, and *GALNT6* in AGS cells stably transfected with mock or *circ-hnRNPU*, and those co-transfected with mNLS form of *NONO* or treated with actinomycin D (5 μg/ml) as indicated. **i**, Western blot assay indicating the expression of hnRNPU, GALNT2, and GALNT6 in AGS cells stably transfected with mock or *circ-hnRNPU*, and those co-transfected with mNLS form of *NONO.* Student’s *t* test or analysis of variance compared the difference in **a**-**h**. *P < 0.05, **P < 0.01. Data are shown as mean ± s.e.m. (error bars) or representative of three independent experiments in **a**-**i**

We further investigated the interplay between *circ-hnRNPU* and NONO in regulating gene expression. In dual-luciferase assay with a reporter containing three canonical c-Myc binding sites, ectopic expression or knockdown of *circ-hnRNPU* prevented the increase or decrease of c-Myc transactivation induced by over-expression or silencing of *NONO* or *c-Myc* in MKN-45, AGS, NCI-N87, HeLa, and PC-3 cells, respectively (Fig. 5a, Additional file 1: Fig. S4a-c). Stable over-expression or knockdown of *circ-hnRNPU* led to decrease or increase in c-Myc binding to *GALNT6* and *MGAT1* promoters, which was prevented by over-expression or silencing of *NONO* or *c-Myc* (Fig. 5b, Additional file 1: Fig. S4d). Consistently, there was reduction or elevation in promoter activity, transcript levels, or protein expression of *GALNT6* and *MGAT1* in cancer cells stably transfected with *circ-hnRNPU* or sh-circ-hnRNPU #1, along with altered expression of downstream O-glycosylated proteins FN1 [29] and GLUT1 [30], which were abolished by ectopic expression or knockdown of *NONO* or *c-Myc* (Fig. 5c, d, Additional file 1: Fig. S4e-g).

To explore the roles of cytoplasmic NONO, the altered genes in RNA-seq assay was further over-lapped with NONO-binding transcripts derived from CLIP-seq (GSE90650) [31], which indicated 29 potential targets, including *hnRNPU*, *GALNT2*, and *GALNT6* (Fig. 5e). Validating RIP assay revealed endogenous enrichment of NONO on 3'-untranslated region (UTR) of *hnRNPU*, *GALNT2*, and *GALNT6* in AGS cells (Fig. 5e). In dual-luciferase assay with a reporter containing four canonical NONO binding sites locating downstream of *Renilla* luciferase, over-expression or silencing of *circ-hnRNPU* reduced or facilitated the 3'-UTR regulatory activity of NONO in MKN-45, AGS, NCI-N87, HeLa, or PC-3 cells, respectively (Fig. 5f, Additional file 1: Fig. S5a). Consistently, stable ectopic expression or knockdown of *circ-hnRNPU* decreased or increased the NONO enrichment on 3'-UTR and mRNA stability of downstream targets (*hnRNPU*, *GALNT2*, and *GALNT6*), resulting in their down-regulation or up-regulation in AGS, NCI-N87, HeLa, or PC-3 cells, while tranfection of NLS-mutant *NONO* or shRNA targeting *NONO* restored these alterations (Fig. 5g-i, Additional file 1: Fig. S5b-e). These results showed that *circ-hnRNPU* inhibited dual NONO activity in regulating glycosyltransferase and *hnRNPU* expression in gastric cancer.

### *Circ-hnRNPU* inhibits glycosylation, tumorigenesis, and aggressiveness of gastric cancer by repressing NONO activity

**Fig. 6:**
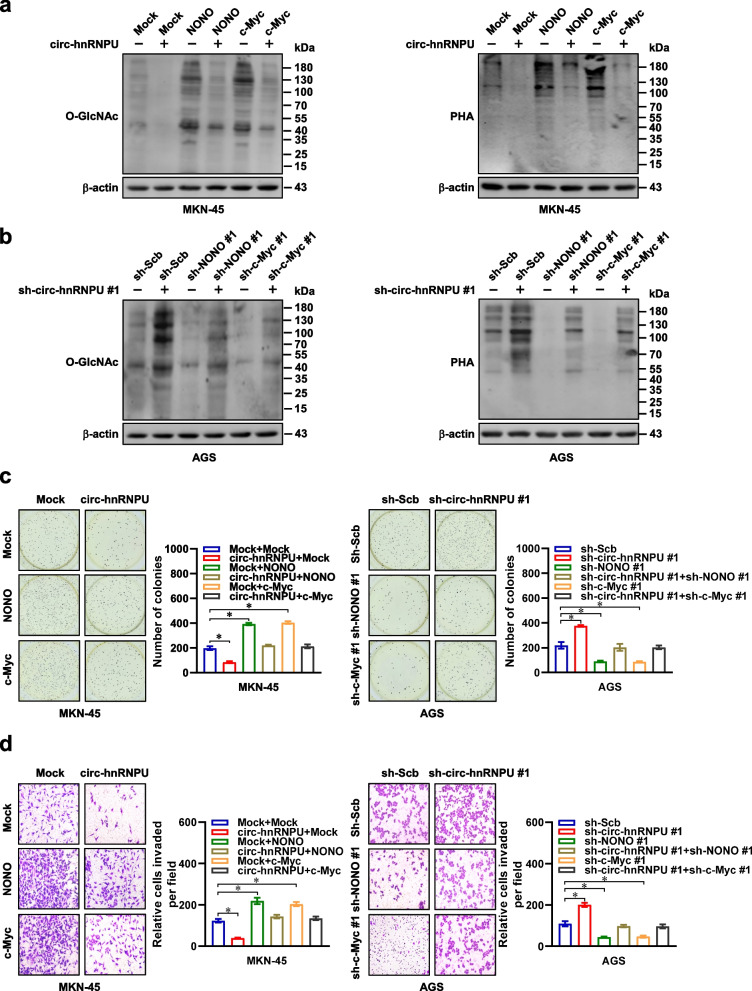
*Circ-hnRNPU* inhibits protein glycosylation, growth, and invasion of gastric cancer cells by repressing NONO or c-Myc activity. **a** and **b**, Western blot assay showing the levels of O- and N-glycosylation in MKN-45 and AGS cells stably transfected with empty vector (mock), *circ-hnRNPU*, scramble shRNA (sh-Scb), or sh-*circ-hnRNPU* #1, and those co-transfected with *NONO*, *c-Myc*, sh-NONO #1, or sh-c-Myc #1. **c** and **d**, Representative images (left) and quantification (right) of soft agar (**c**) and matrigel invasion (**d**) assays indicating the anchorage-independent growth and invasion of MKN-45 and AGS cells stably transfected with mock, *circ-hnRNPU*, sh-Scb, or sh-*circ-hnRNPU* #1, and those co-transfected with *NONO*, *c-Myc*, sh-NONO #1, or sh-c-Myc #1. Student’s *t* test compared the difference in **c** and **d**. *P < 0.05. Data are shown as mean ± s.e.m. (error bars) or representative of three independent experiments in **a**-**d**

**Fig. 7:**
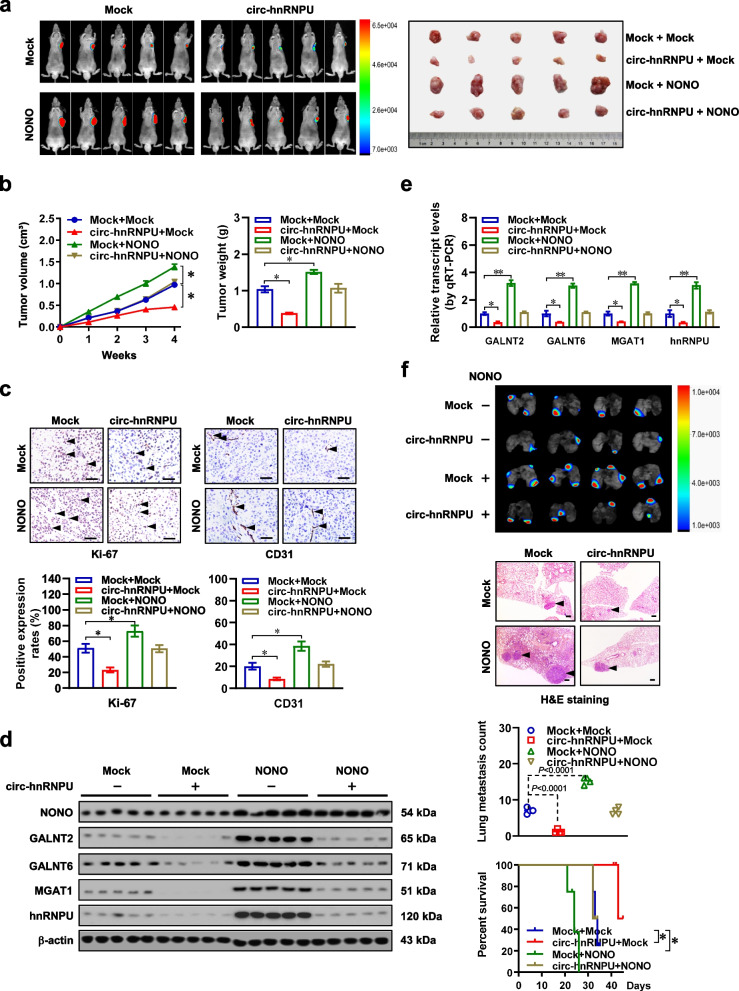
*Circ-hnRNPU* inhibits gastric cancer progression by repressing NONO activity in vivo. **a** and **b**, Representative imagines (**a**), in vivo growth curve (**b**, left panel), and weight at the end points (**b**, right panel) of xenograft tumors formed by subcutaneous injection of AGS cells stably transfected with empty vector (mock) or *NONO*, and those co-transfected with *circ-hnRNPU* into the dorsal flanks of nude mice (*n* = 5 for each group). **c**, Representative images (upper panel) and quantification (lower panel) of immunohistochemical staining showing the expression of Ki-67 and CD31 (arrowheads) within xenograft tumors formed by hypodermic injection of AGS cells stably transfected with mock or *NONO*, and those co-transfected with *circ-hnRNPU* (*n* = 5 for each group). Scale bars: 50 μm. **d** and **e**, Western blot (d) and real-time qRT-PCR (e, normalized to *β-actin*) assays indicating the levels of *NONO*, *GALNT2*, *GALNT6*, *MGAT1*, and *hnRNPU* in xenograft tumors formed by hypodermic injection of AGS cells stably transfected with mock or *NONO*, and those co-transfected with *circ-hnRNPU* (*n* = 5 for each group). **f**, Representative images (upper panel), H&E staining (arrowheads), and quantification of lung metastatic colonization (middle panels), as well as Kaplan–Meier curves (lower panel) of nude mice treated with tail vein injection of AGS cells stably transfected with mock or *NONO*, and those co-transfected with *circ-hnRNPU* (*n* = 4 for each group). Scale bar: 100 μm. Analysis of variance or Student’s *t* test compared the difference in **b**, **c**, **e**, and **f**. Log-rank test for survival comparison in **f**. *P < 0.05, **P < 0.01. Data are shown as mean ± s.e.m. (error bars) in **b**, **c**, **e**, and **f**

Next, we further investigated the functions of *circ-hnRNPU* and *NONO* in gastric cancer progression. Ectopic expression or silencing of *NONO* or *c-Myc* prevented the reduction or elevation of O-glycosylation and N-glycosylation in MKN-45 and AGS cells with stable over-expression or knockdown of *circ-hnRNPU* (Fig. 6a, b). Notably, ectopic expression or silencing of *NONO* or *c-Myc* partially restored the decrease or increase of growth and invasion of MKN-45 and AGS cells induced by over-expression or knockdown of *circ-hnRNPU* (Fig. 6c, d). Consistently, stable transfection of *NONO* promoted the growth, weight, Ki-67 proliferation index, CD31-positive microvessels, target gene expression, and O-glycosylation and N-glycosylation levels of subcutaneous xenograft tumors formed by AGS cells in nude mice, which were attenutaed by *circ-hnRNPU* over-expression (Fig. 7a-e, Additional file 1: Fig. S5f, g). Athymic nude mice treated with tail vein injection of AGS cells stably over-expressing *NONO* displayed more lung metastatic colonies and poorer overall survival probability, which were attenuated by ectopic expression of *circ-hnRNPU* (Fig. 7f). These data suggested that *circ-hnRNPU* inhibited glycosylation, tumorigenesis, and aggressiveness of gastric cancer by repressing NONO activity.

### Therapeutic effects of lentivirus-mediated *circ-hnRNPU* over-expression in vivo

**Fig. 8:**
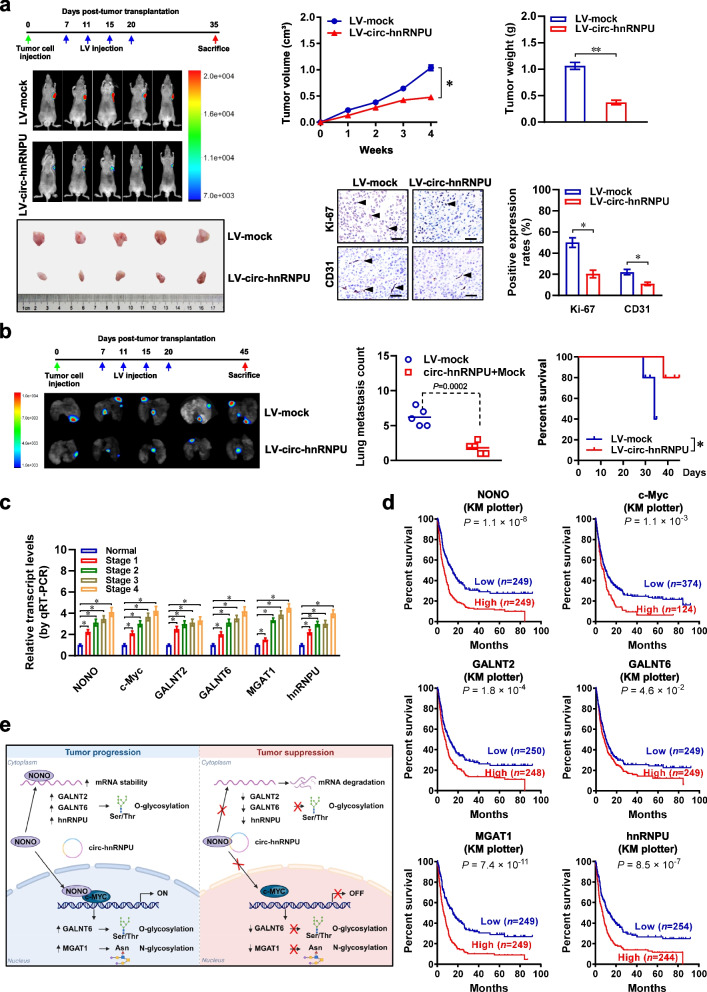
Therapeutic effects of lentivirus-mediated *circ-hnRNPU* over-expression in vivo. **a**, Representative images (left panels), in vivo growth curve (upper middle panel), weight at the end points (upper right panel), and immunohistochemical staining of Ki-67 and CD31 (lower right panels, arrowheads) of xenograft tumors formed by subcutaneous injection of MKN-45 cells into dorsal flanks of nude mice (*n* = 5 for each group) that received intravenous administration of lentiviral empty vector (LV-mock) or *circ-hnRNPU* (LV-circ-hnRNPU) as indicated. Scale bar: 100 μm. **b**, Representative images (left panel), quantification of lung metastatic colonization (middle panel), and Kaplan–Meier curves (right panel) of nude mice treated with tail vein injection of MKN-45 cells and LV-mock or *circ-hnRNPU* (LV-circ-hnRNPU) as indicated. Scale bar: 100 μm. **c**, Real-time qRT-PCR (normalized to *β-actin*) assay indicating the levels of *NONO*, *c-Myc*, and target genes in normal gastric mucosa (*n* = 40) and gastric cancer tissues (*n* = 81). **d**, Kaplan–Meier curves showing the overall survival of gastric cancer cases derived from KM Plotter database (http://kmplot.com/analysis) with low or high expression of *NONO* (cutoff value = 10.31), *c-Myc* (cutoff value = 10.63), *GALNT2* (cutoff value = 8.36), *GALNT6* (cutoff value = 9.14), *MGAT1* (cutoff value = 9.13), or *hnRNPU* (cutoff value = 5.21). **e**, The mechanisms underlying tumor suppressive roles of *circ-hnRNPU*: as a circRNA derived from *hnRNPU*, *circ-hnRNPU* directly interacts with NONO to induce its cytoplasmic retention, which restrains nuclear NONO-facilitated c-Myc transactivation or cytoplasmic NONO-enhanced mRNA stability of glycosyltransferases and *hnRNPU*, resulting in repression of glycosylation and cancer progression. Analysis of variance or Student’s *t* test compared the difference in **a** and **b**. Mann–Whitney U test compared the difference in **c**. Log-rank test for survival comparison in **b** and **d**. *P < 0.05, **P < 0.01. Data are shown as mean ± s.e.m. (error bars) in **a**-**c**

To assess therapeutic potential of lentivirus-mediated *circ-hnRNPU* over-expression, nude mice were treated with subcutaneous or tail vein injection of MKN-45 cells stably expressing red fluorescent protein (Fig. 8a). Intravenous administration of lentivirus carrying *circ-hnRNPU* resulted in significant reduction of growth, tumor weight, Ki-67 proliferation index, and CD31-positive intratmoral microvessels of subcutaneous xenograft tumors (Fig. 8a). The intratumoral expression of *circ-hnRNPU*, *GALNT2*, *GALNT6*, *MGAT1*, and *hnRNPU* was significantly altered in xenograft tumors treated with lentivirus carrying *circ-hnRNPU* (Additional file 1: Fig. S6a, b), accompanied by reduction in O- and N-glycosylation levels (Additional file 1: Fig. S6c). In therapeutic experiments on cancer metastasis, intravenous administration of lentivirus carrying *circ-hnRNPU* decreased the lung metastatic colonies and increased the survival probability of nude mice treated with tail vein injection of MKN-45 cells (Fig. 8b, Additional file 1: Fig. S6d).

In 81 primary gastric cancer specimens, the expression of *NONO*, *c-Myc*, and target genes were increased, especially in those with advanced clinical stages (Fig. 8c). There was significantly elevated cytoplasmic NONO expression in gastric cancer specimens with high *circ-hnRNPU* levels (Additional file 1: Fig. S7a). In these gastric cancer cases, the expression of *circ-hnRNPU*, *NONO*, or *c-Myc* was negatively or positively correlated with that of target genes *GALNT2*, *GALNT6*, *MGAT1*, or *hnRNPU* (Additional file 1: Fig. S7b). In a public gastric cancer dataset derived from Kaplan–Meier Plotter (http://kmplot.com/analysis) database, gastric cancer patients with higher expression of *NONO* (*P* = 1.1 × 10^–8^), *c-Myc* (*P* = 1.1 × 10^–3^), *GALNT2* (*P* = 1.8 × 10^–4^), *GALNT6* (*P* = 4.6 × 10^–2^), *MGAT1* (*P* = 7.4 × 10^–11^), or *hnRNPU* (*P* = 8.5 × 10^–7^) had lower survival possibility (Fig. 8d). Moreover, high expression of *NONO* or *c-Myc* was correlated with poor survival of patients with adrenocortical carcinoma, bladder cancer, breast cancer, colon cancer, esophagus cancer, glioblastoma, lung cancer, ovarian cancer, or renal cancer (Additional file 1: Fig. S8). These results suggested that lentivirus-mediated *circ-hnRNPU* over-expression inhibited tumorigenesis and aggressiveness in vivo.

## Discussion

HnRNPU is a multi-potent nuclear protein regulating transcriptional activation, RNA processing, or genome organization [15], and contributes to drug resistance and progression of cancer [32]. In this study, we identify one circRNA derived from hnRNPU (*circ-hnRNPU*) as a tumor suppressor significantly down-regulated in gastric cancer, which inhibits the glycosylation, proliferation, invasion, and migration of gastric cancer cells. Human O-linked and N-linked glycosylation are catalyzed by glycosyltransferases [2]. As one member of GALNT family, *GALNT2* is dysregulated in many types of cancers, and plays tumor suppressive or promoting roles via regulating protein O-glycosylation, such as epidermal growth factor receptor [33] and AXL receptor tyrosine kinase [34]. In non-small cell lung cancer, GALNT2 modifies the O-glycosylation of integrin α5 to activate signal pathways essential for tumorigenesis and aggressiveness [35]. Previous studies indicate that *GALNT6* catalyzes the transfer of N-acetylgalactosamine (GalNAc) to serine/threonine residues of substrate protein, and is responsible for mucin-type O-glycosylation [2]. In breast cancer, *GALNT6* is related with poor prognosis of patients, and promotes the proliferation, epithelial-mesenchymal transition, migration, and invasion of cancer cells via O-glycosylation and stabilization of fibronectin [29] or mucin 1 [36]. MGAT1, one protein contributing to N-glycan synthesis, promotes the proliferation and migration of glioma or Wilms' tumor cells via N-glycosylation and up-regulation of GLUT1 [30] or mucin 3A [37], while inhibition of *MGAT1* expression suppresses the growth and metastasis of breast cancer [38]. In the current study, we found that *circ-hnRNPU* physically interacted with and induced cytoplasmic retention of NONO protein, which inhibited nuclear NONO-facilitated c-Myc transactivation or cytoplasmic NONO-enhanced mRNA stability of glycosyltransferases (*GALNT2*, *GALNT6*, *MGAT1*) and *hnRNPU*, resulting in repression of glycosylation, tumorigenesis, and aggressiveness (Fig. 8e). Interestingly, recent studies show that *hnRNPU*-derived *hsa_circ_0017272* is able to encode a protein consisting of 603 aa, which promotes the proliferation of multiple myeloma cells via regulating exon skipping of S-phase kinase associated protein 2 and stabilizing c-Myc protein [39]. These findings indicate that *hnRNPU*-derived circRNAs exert diverse functions in tumorigenesis and aggressiveness. Our rescue studies indicated that *circ-hnRNPU* possessed tumor suppressive properties, at least in part, via repressing NONO activity, suggesting its potential as a therapeutic target against cancers.

NONO/p54nrb is a member of the *Drosophila* behaviour/human splicing (DBHS) family [40]. To date, three human DBHS protein paralogues have been reported, including splicing factor proline and glutamine rich (SFPQ) [41], NONO, and paraspeckle component 1 (PSPC1) [42], which are consisted of highly conservative N-terminal RRMs, a NONA/paraspeckle domain (NOPS), and a C-terminal coiled-coil [40]. NONO functions as a multipurpose molecular scaffold mediating extensive protein–protein and protein-nucleic acid interactions [43], and is engaged in many regulatory aspects of gene expression, such as transcriptional repression [44] or activation [45] and post-transcriptional processing [46]. In addition, *NONO* participates in paraspeckle formation [47], DNA double-strand break repair [48], cell cycle and circadian rhythm [49], and is closely related to cancer progression [40, 47]. By using cell lines of gastric cancer, cervical cancer, and prostate cancer, we found that within the nucleus, NONO served as a co-factor essential for c-Myc transactivation in regulating expression of *GALNT6* and *MGAT1*. Meanwhile, mRNA stability of *GALNT2*, *GALNT6*, and *hnRNPU* was maintained by cytoplasmic NONO, suggesting the dual NONO activity in regulating c-Myc transactivation and mRNA stabilization essential for protein glycosylation and cancer progression.

C-Myc is an important oncoprotein contributing to 30–50% of human malignancies [50]. As a transcription factor, c-Myc regulates up to 15% of human genes through forming a transcription complex with Max to bind specific DNA consensus sequence CANNTG (known as E-box), and is critical for cell proliferation, metabolism, and somatic cell reprogramming [50]. However, the exact functions and regulation of c-Myc activity in protein glycosylation within cancers remain largely unknown. In this study, we found that c-Myc directly facilitated the transcription of *GALNT6* and *MGAT1* in gastric cancer cells, leading to O-glycosylation or N-glycosylation and stabilization of FN1 and GLUT1 protein, respectively. Of note, c-Myc also directly facilitated the transcription of *GLUT1*, an established downstream gene involved in glycolytic process [51], suggesting its multiple action modes in regulating glycolytic gene expression. Within the N-terminal region, transactivational domain (TAD) is involved in affecting c-Myc activity via recruiting a number of co-regulators, such as high mobility group box protein 1 [52] or ribosomal protein L11 [53]. Our results indicated that carboxyl-terminal domain of NONO protein interacted with TAD of c-Myc, which facilitated the binding of c-Myc to target gene promoters. Meanwhile, *circ-hnRNPU* interacted with RRM1 domain of NONO to stimulate its cytoplasmic aggregation, which attenuated c-Myc transactivation, O-glycosylation, and N-glycosylation in cancer cells. Consistent with previous studies showing cytoplasmic aggregation of NONO or homologous SFPQ triggered by molecular partner [54] or zinc ion [55], our findings indicate that *circ-hnRNPU* may inactivate nuclear localization signal of NONO to attenuate its co-activator role in c-Myc-mediated protein glycosylation.

## Conclusions

In summary, we have demonstrated that *circ-hnRNPU* is down-regulated in gastric cancer, and closely related to favorable outcome of patients. *Circ-hnRNPU* directly interacts with NONO to induce its cytoplasmic aggregation, which further restrains c-Myc transactivation in driving *GALNT6* and *MGAT1* expression. Importantly, as a RBP, cytoplasmic NONO stabilizes *GALNT2*, *GALNT6*, and *hnRNPU* mRNA in gastric cancer cells, which is also repressed by *circ-hnRNPU*. Meanwhile, the roles of *NONO* and *c-Myc* in regulating O- and N-glycosylation of other proteins warrant further investigation. Exploration of other protein partners is helpful for elucidating additional functions of *circ-hnRNPU* during cancer progression. We believe that this study extends our knowledge about the regulation of glycosyltransferase and *hnRNPU* expression by circRNA and its protein partner, and suggests that *circ-hnRNPU*/NONO/c-Myc axis may be a potential therapeutic target for gastric cancer.

### Supplementary Information

**Additional file 1: Figure S1.** Expression profiles of *circ-hnRNPU* in cancer cells. **Figure S2.** *circ-hnRNPU* inhibits the growth and invasion of gastric cancer cells. **Figure S3.** Interaction domains between NONO and c-Myc. **Figure S4.** *circ-hnRNPU* represses NONO-facilitated c-Myc transactivation in regulating glycosyltransferase expression. **Figure S5.** *circ-hnRNPU* inhibits cytoplasmic NONO-facilitated mRNA stability of glycosyltransferases. **Figure S6.** Lentivirus-mediated *circ-hnRNPU* over-expression represses glycosylation and lung metastasis. **Figure S7.** Expression correlation of *circ-hnRNPU*, *NONO*, *c-Myc*, and target genes in gastric cancer tissues. **Figure S8.** Kaplan–Meier curves of *NONO* and *c-Myc* in multiple cancers. **Table S1.** Primer sets used for RT-PCR, qPCR, ChIP, and RIP. **Table S2.** Oligonucleotide sets used for short hairpin RNAs, probe, or guide DNA. **Table S3.** Oligonucleotide sets used for constructs. **Table S4.** Mass spectrometry analysis of proteins pulled down by *circ-hnRNPU*

## Data Availability

The RNA-seq data have been deposited in Gene Expression Omnibus (GEO) repository (https://www.ncbi.nlm.nih.gov/geo), under accession number GSE246847. Public microarray datasets for analyzing association of gene expression with survival of cancer patients are available from Kaplan–Meier Plotter (https://kmplot.com/analysis), The Cancer Genome Atlas (TCGA, https://portal.gdc.cancer.gov), or GEO database (accession number GSE41258, GSE11595, GSE13213, GSE9891, and GSE3538). The ChIP-seq or CLIP-seq data for analyzing c-Myc or NONO peak are obtained from GEO database (accession number: GSE51334 and GSE90650). The data supporting the conclusions of this article are presented within article and its additional files.

## Abbreviations

BiFC: Bimolecular fluorescence complementation
ChIP: Chromatin immunoprecipitation
circRNA: Circular RNA
co-IP: Co-immunoprecipitation
DAPI: 4′,6-Diamidino-2-phenylindole
DIG: Digoxigenin
EMSA: Electrophoretic mobility shift assay
GALNT2: Polypeptide N-acetylgalactosaminyltransferase 2
GALNT6: Polypeptide N-acetylgalactosaminyl-transferase 6
GLUT1: Glucose transporter 1
MGAT1: Alpha-1,3-mannosyl-glycoprotein 2-beta-N-acetylgluco-saminyltransferase
NLS: Nuclear localization signal
NONO: Non-POU domain containing octamer binding
RIP: RNA immunoprecipitation
shRNA: Short hairpin RNA
ST6GAL1: ST6 beta-galactoside alpha-2,6-sialyltransferase 1
