# Supplementary material for: circ-hnRNPU inhibits NONO-mediated c-Myc transactivation and mRNA stabilization essential for glycosylation and cancer progression
Source: J Exp Clin Cancer Res. 2023 Nov 23;42:313. doi: 10.1186/s13046-023-02898-5 (PMC10666356; doi:10.1186/s13046-023-02898-5)
Supplement: Supplementary file 1 — Additional file 1: Figure S1. Expression profiles of circ-hnRNPU in cancer cells. Figure S2. circ-hnRNPU inhibits the growth and invasion of gastric cancer cells. Figure S3. Interaction domains between NONO and c-Myc. Figure S4. circ-hnRNPU represses NONO-facilitated c-Myc transactivation in regulating glycosyltransferase expression. Figure S5. circ-hnRNPU inhibits cytoplasmic NONO-facilitated mRNA stability of glycosyltransferases. Figure S6. Lentivirus-mediated circ-hnRNPU over-expression represses glycosylation and lung metastasis. Figure S7. Expression correlation of circ-hnRNPU, NONO, c-Myc, and target genes in gastric cancer tissues. Figure S8. Kaplan–Meier curves of NONO and c-Myc in multiple cancers. Table S1. Primer sets used for RT-PCR, qPCR, ChIP, and RIP. Table S2. Oligonucleotide sets used for short hairpin RNAs, probe, or guide DNA. Table S3. Oligonucleotide sets used for constructs. Table S4. Mass spectrometry analysis of proteins pulled down by circ-hnRNPU [file 13046_2023_2898_MOESM1_ESM.pdf]

Supporting Information

circ-hnRNPU inhibits NONO-mediated c-Myc transactivation and mRNA stabilization essential for glycosylation and cancer progression

Li et al.

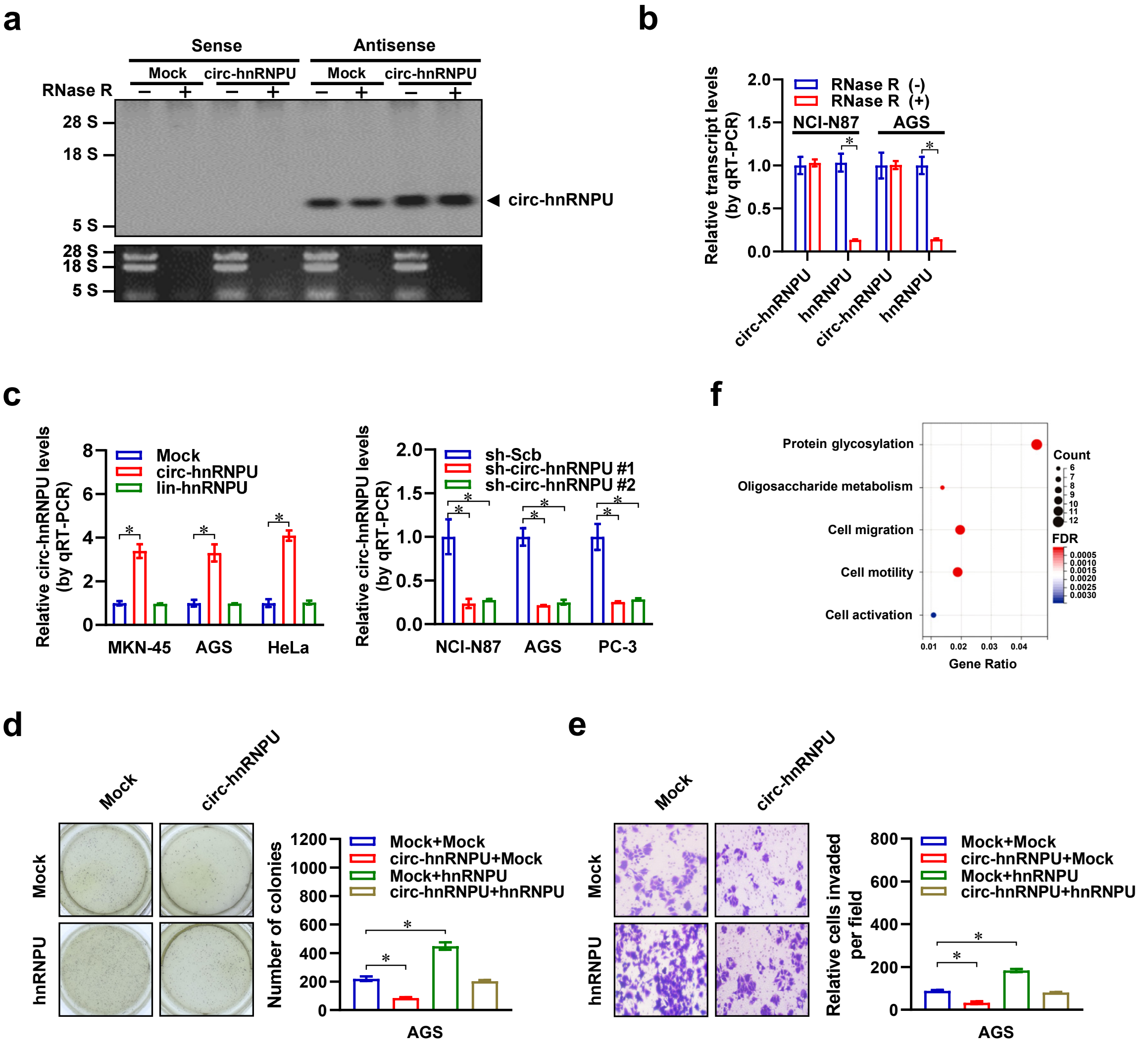

Figure S1. Expression profiles of *circ-hnRNPU* in cancer cells. a, Northern blot using a junction-specific sense or antisense probe indicating the endogenous and exogenous existence of *circ-hnRNPU* in AGS cells transfected with empty vector (mock) or *circ-hnRNPU*, and those treated with RNase R (3 U/mg). **b**, Real-time qRT-PCR assay showing the levels (normalized to *β-actin*, n=5) of *circ-hnRNPU* and *hnRNPU* in NCI-N87 and AGS cells treated with RNase R (3 U/mg). **c**, Real-time qRT-PCR assay showing the levels (normalized to *β-actin*, *n*=5) of *circ-hnRNPU* in MKN-45, AGS, HeLa, NCI-N87, and PC-3 cells stably transfected with mock, *circ-hnRNPU*, linear *circ-hnRNPU* (*lin-hnRNPU*), scramble shRNA (sh-Scb), sh-*circ-hnRNPU* #1, or sh-*circ-hnRNPU* #2. **d** and **e**, Representative images (left panel) and quantification (right panel) of soft agar (d) and matrigel invasion (e) assays indicating the anchorage-independent growth and invasion of AGS cells stably transfected with mock, *circ-hnRNPU*, or *hnRNPU*. **f**, Gene ontology analysis showing the involved biological processes of altered genes in MKN-45 and AGS cells stably transfected with mock or *circ-hnRNPU*. Student's *t* test compared the difference in **b**-**e**. *P<0.05. Data are shown as mean ± s.e.m. (error bars) or representative of three independent experiments in **a**-**e**.

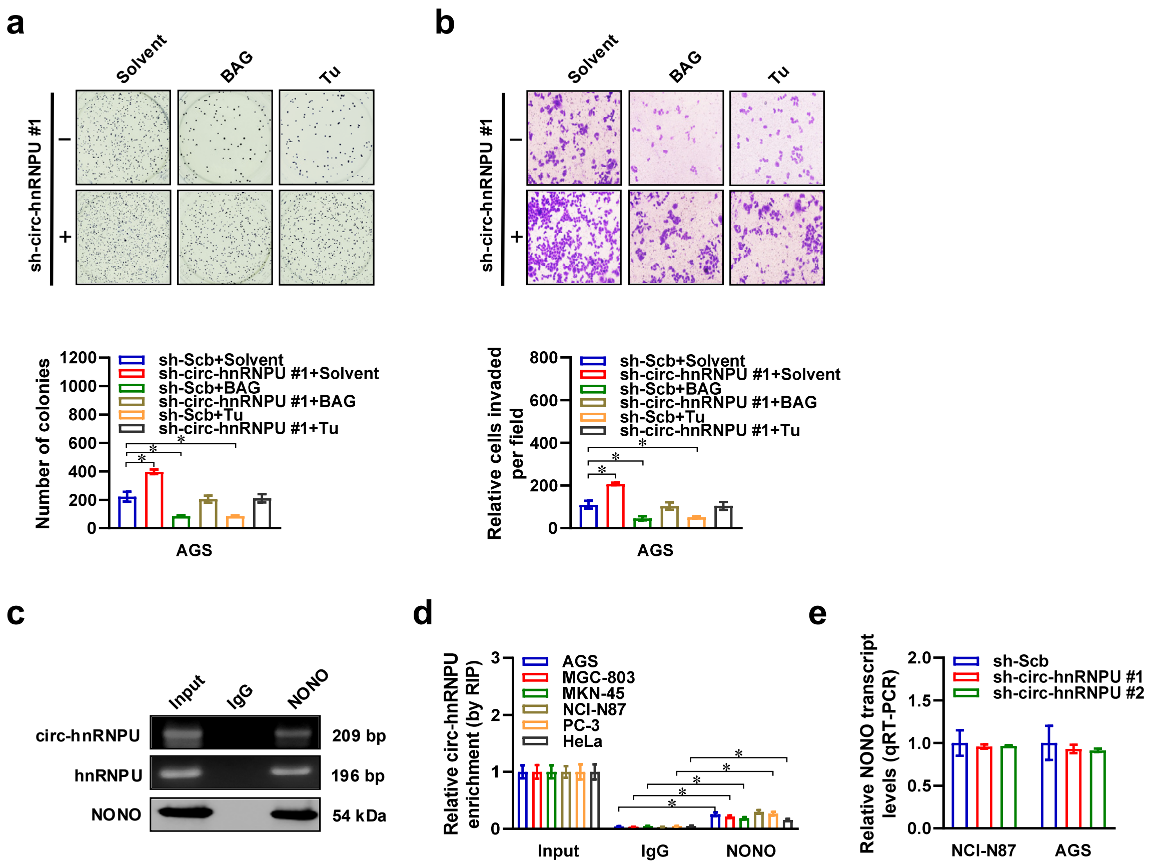

Figure S2. ***circ-hnRNPU* inhibits the growth and invasion of gastric cancer cells. a** and **b**, Representative images (upper panel) and quantification (lower panel) of soft agar (a) and matrigel invasion (b) assays indicating the anchorage-independent growth and invasion of AGS cells stably transfected with scramble shRNA (sh-Scb) or sh-*circ-hnRNPU* #1, and those treated with benzyl-α-GalNAc (BAG, 2.0 mmol/L) or tunicamycin (Tu, 0.5 μg/ml) for 24 hours. **c**, RIP (upper panel) and western blot (lower panel) assays using NONO antibody showing endogenous interaction of NONO with *circ-hnRNPU* or *hnRNPU* mRNA in NCI-N87 cells. **d**, RIP and real-time qRT-PCR (normalized to input, n=5) assays indicating endogenous binding of NONO to *circ-hnRNPU* in cultured cancer cell lines. **e**, Real-time qRT-PCR assay showing the *NONO* levels (normalized to *β-actin*, n=5) in NCI-N87 and AGS cells stably transfected with sh-Scb, sh-*circ-hnRNPU* #1, or sh-*circ-hnRNPU* #2. Student's t test compared the difference in **a**, **b**, **d** and **e**. *P<0.05. Data are shown as mean ± s.e.m. (error bars) or representative of three independent experiments in **a**-**e**.

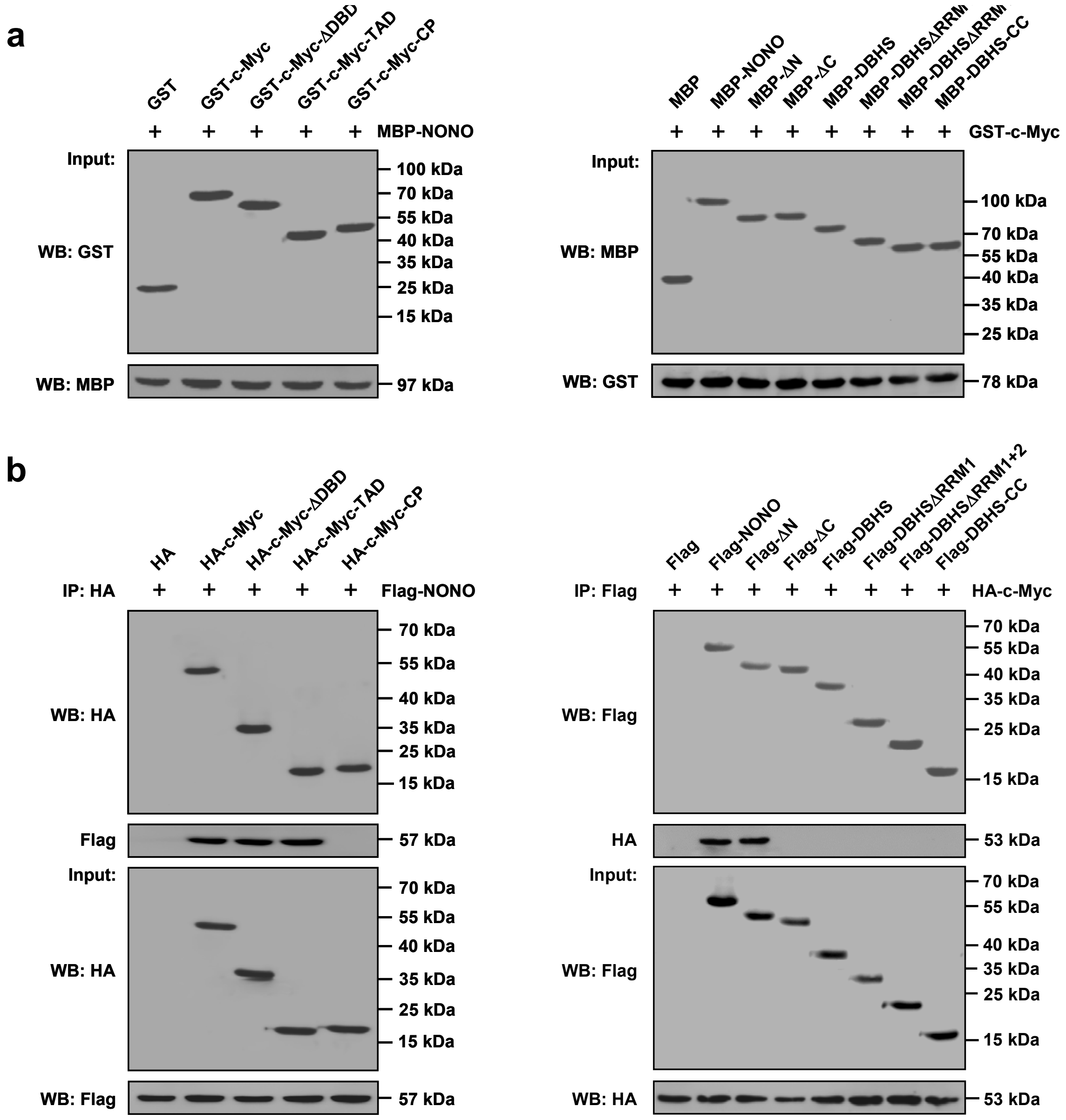

Figure S3. Interaction domains between NONO and c-Myc. a, Western blot assays showing the levels of full-length or truncated GST-tagged c-Myc and MBP-tagged NONO proteins in input groups. **b**, Co-IP and western blot assays indicating the interaction between NONO and c-Myc in AGS cells transfected with full-length or truncations of Flag-tagged *NONO* and HA-tagged *c-Myc* constructs. Data are shown as representative of three independent experiments in **a** and **b**.

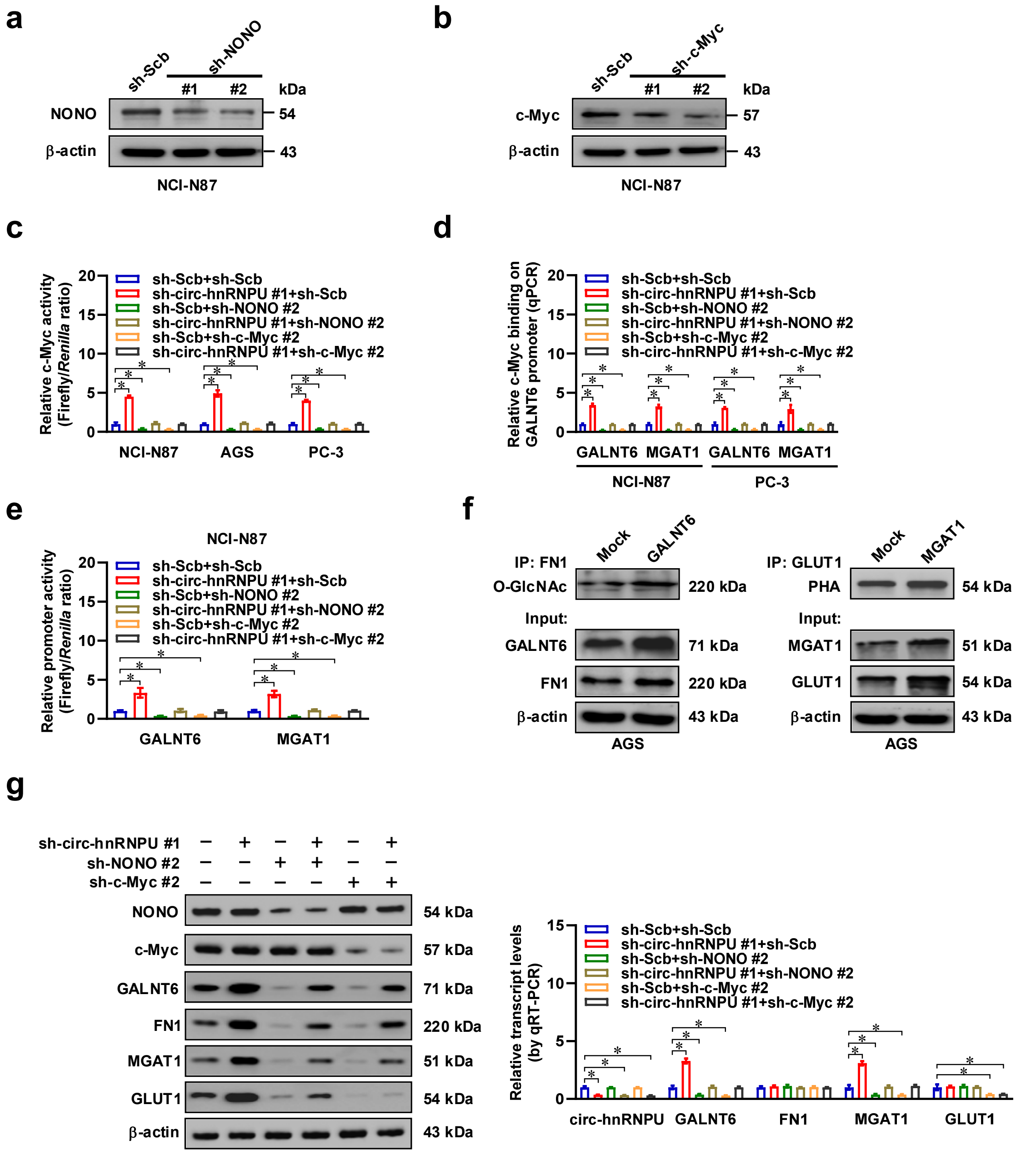

Figure S4. ***circ-hnRNPU* represses NONO-facilitated c-Myc transactivation in regulating glycosyltransferase expression. a** and **b**, Western blot assay showing the levels of NONO or c-Myc in NCI-N87 cells stably transfected with scramble shRNA (sh-Scb), sh-NONO, or sh-c-Myc. **c** and **d**, Dual-luciferase assay using a reporter containing three canonical c-Myc binding sites (c), ChIP and qPCR (d) assays showing the transactivation and enrichment of c-Myc on *GALNT6 or MGAT1* promoter in NCI-N87, AGS, and PC-3 cells stably transfected with sh-Scb or sh-*circ-hnRNPU* #1, and those co-transfected with sh-NONO #2 or sh-c-Myc #2. **e**, Dual-luciferase assay revealing the promoter activity of *GALNT6* or *MGAT1* in NCI-N87 cells stably transfected with sh-Scb or sh-*circ-hnRNPU* #1, and those co-transfected with sh-NONO #2 or sh-c-Myc #2. **f**, Co-IP and western blot assays indicating the O-glycosylation, N-glycosylation, and expression of FN1 or GLUT1 in AGS cells transfected with empty vector (mock), *GALNT6*, or *MGAT1*. **g**, Western blot and real-time qRT-PCR (normalized to *β-actin*) assays showing the expression of *circ-hnRNPU*, *GALNT6, MGAT1,* and downstream target (*FN1* and *GLUT1*) levels in NCI-N87 cells stably transfected with sh-Scb or sh-*circ-hnRNPU* #1, and those co-transfected with sh-NONO #2 or sh-c-Myc #2. Student's *t* test compared the difference in **c**-**e** and **g**. $^{*}P<0.05$. Data are shown as mean ± s.e.m. (error bars) or representative of three independent experiments in **a**-**g**.

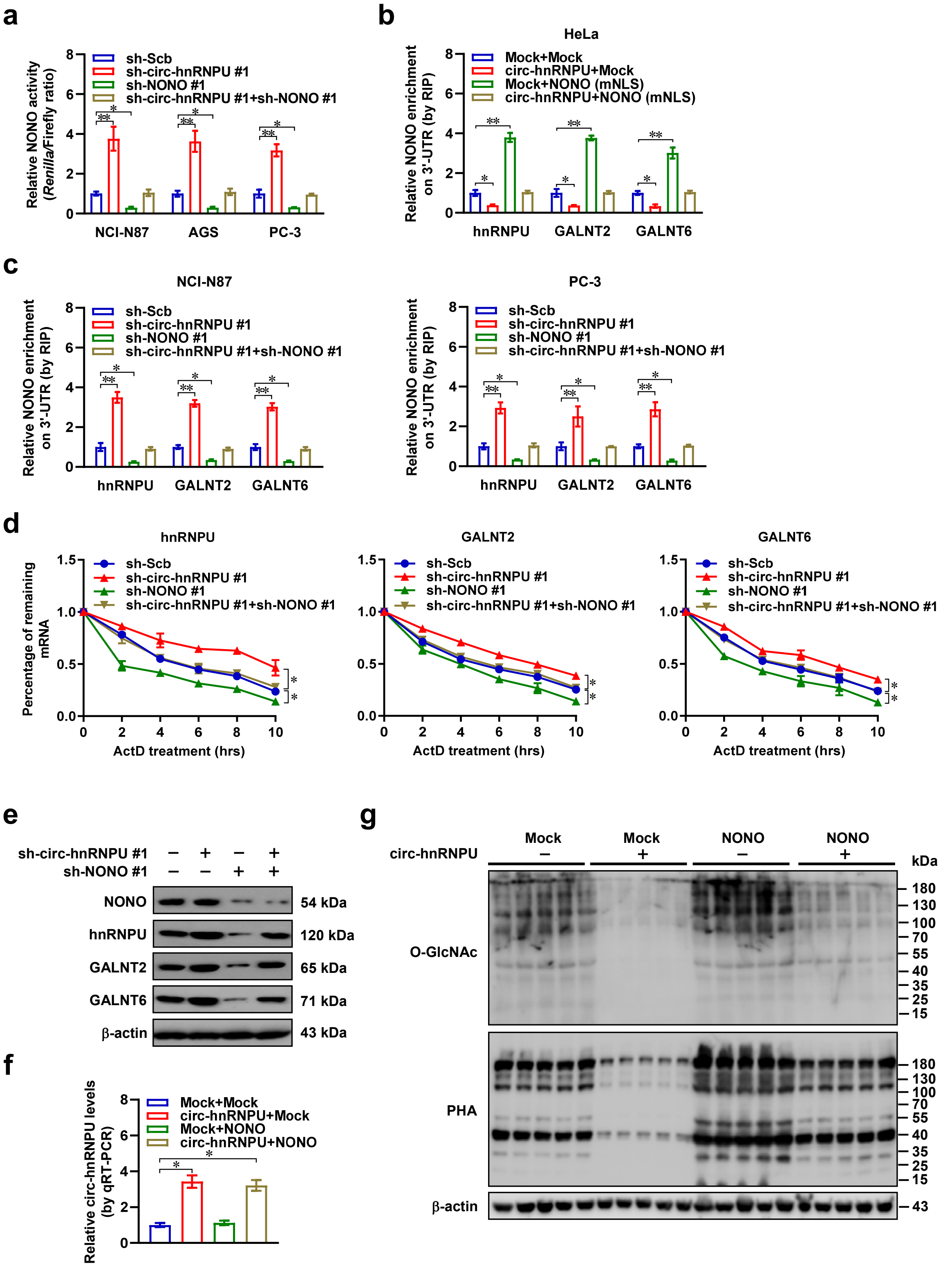

Figure S5. ***circ-hnRNPU*** **inhibits cytoplasmic NONO-facilitated mRNA stability of glycosyltransferases.** **a**-**d**, Dual-luciferase assay using a 3'-UTR reporter containing four canonical NONO binding sites (a), RIP and real-time qRT-PCR (b, c), and real-time qRT-PCR (d, normalized to *β-actin*, *n*=5) assays showing the NONO activity, NONO enrichment on 3'-UTR, and mRNA stability of *hnRNPU*, *GALNT2*, or *GALNT6* in indicated cancer cells, without or with actinomycin D (5 μg/ml) treatment. **e**, Western blot assay showing the levels of hnRNPU, GALNT2, or GALNT6 in NCI-N87 cells stably transfected with sh-Scb or sh-*circ-hnRNPU* #1, and those co-transfected with sh-NONO #1. **f** and **g**, Real-time qRT-PCR (e, normalized to *β-actin*) and western blot (f) assays indicating the levels of *circ-hnRNPU*, O-glycosylation, and N-glycosylation in xenograft tumors formed by hypodermic injection of AGS cells stably transfected with mock or *NONO*, and those co-transfected with *circ-hnRNPU* (*n*=5 for each group). Student's *t* test or analysis of variance compared the difference in **a**-**d** and **f**. **P*<0.05, ***P*<0.01. Data are shown as mean ± s.e.m. (error bars) or representative of three independent experiments in **a**-**e**.

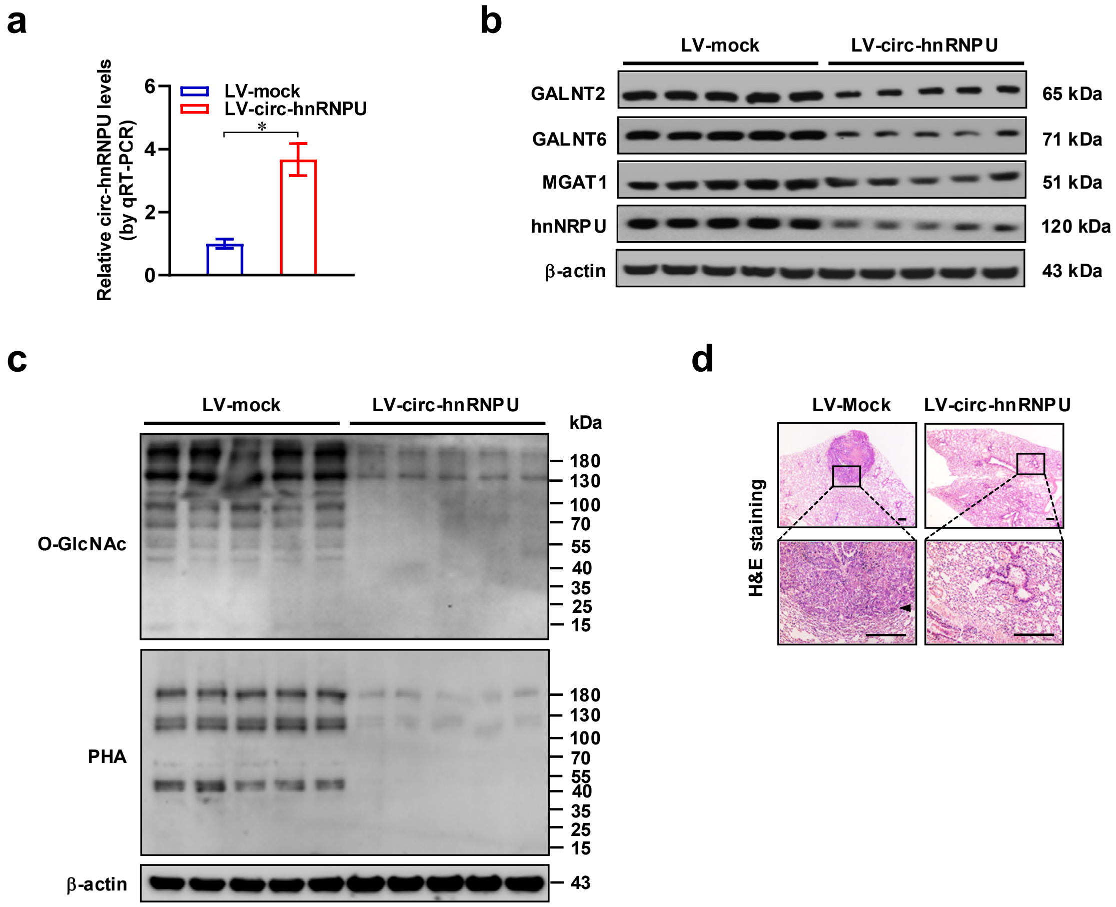

Figure S6. Lentivirus-mediated *circ-hnRNPU* over-expression represses glycosylation and lung metastasis. a and **b**, Real-time qRT-PCR (a, normalized to *β-actin*, *n*=5) and western blot (b) assays showing the expression of *circ-hnRNPU*, *GALNT2*, *GALNT6*, *MGAT1*, and *hnRNPU* in xenograft tumors formed by subcutaneous injection of MKN-45 cells into dorsal flanks of nude mice (*n* = 5 for each group) that received intravenous administration of lentiviral empty vector (LV-mock) or *circ-hnRNPU* (LV-circ-hnRNPU). **c**, Western blot assay revealing the levels of O-glycosylation and N-glycosylation in xenograft tumors formed by subcutaneous injection of MKN-45 cells into dorsal flanks of nude mice (*n* = 5 for each group) that received intravenous administration of LV-mock or LV-circ-hnRNPU. **d**, HE staining showing the lung metastasis (arrowheads) of nude mice treated with tail vein injection of MKN-45 cells and LV-mock or LV-circ-hnRNPU. Scale bar: 100 μm. Student's *t* test compared the difference in **a**. **P*<0.05. Data are shown as mean ± s.e.m. (error bars) in **a**.

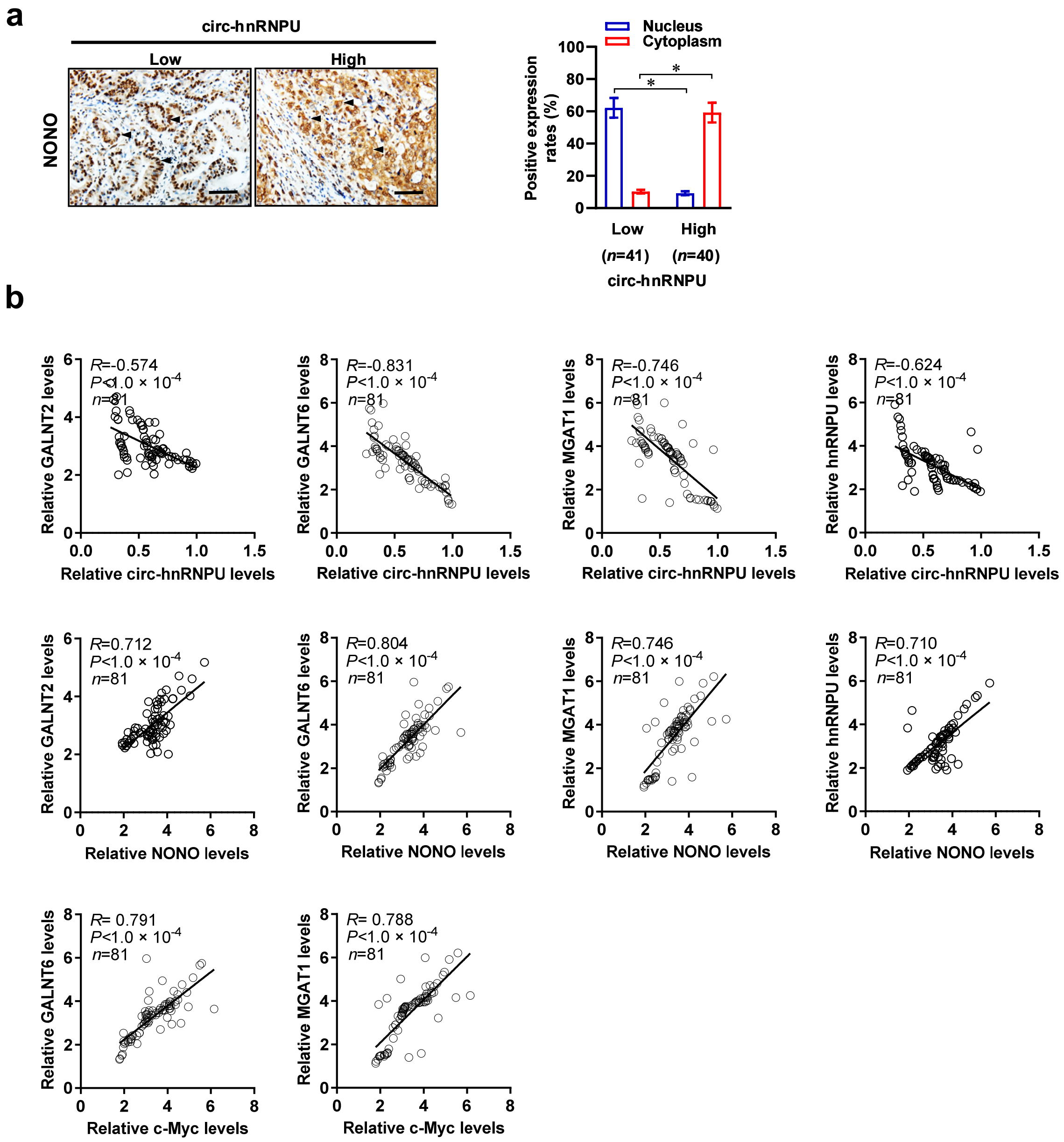

Figure S7. Expression correlation of *circ-hnRNPU*, *NONO*, *c-Myc*, and target genes in gastric cancer tissues. a, Representative images (left panel) and quantification (right panel) of immunohistochemical staining showing the NONO expression (arrowheads) in gastric cancer tissues with low or high levels of *circ-hnRNPU* (cutoff value=0.58). **b**, Expression correlation of *circ-hnRNPU*, *NONO*, *c-Myc*, and target genes (*GALNT2*, *GALNT6*, *MGAT1*, or *hnRNPU*) in primary gastric cancer tissues (n=81). Student's t test compared the difference in **a**. Pearson's correlation coefficient assay in **b**. *P<0.05. Data are shown as mean ± s.e.m. (error bars) in **a**.

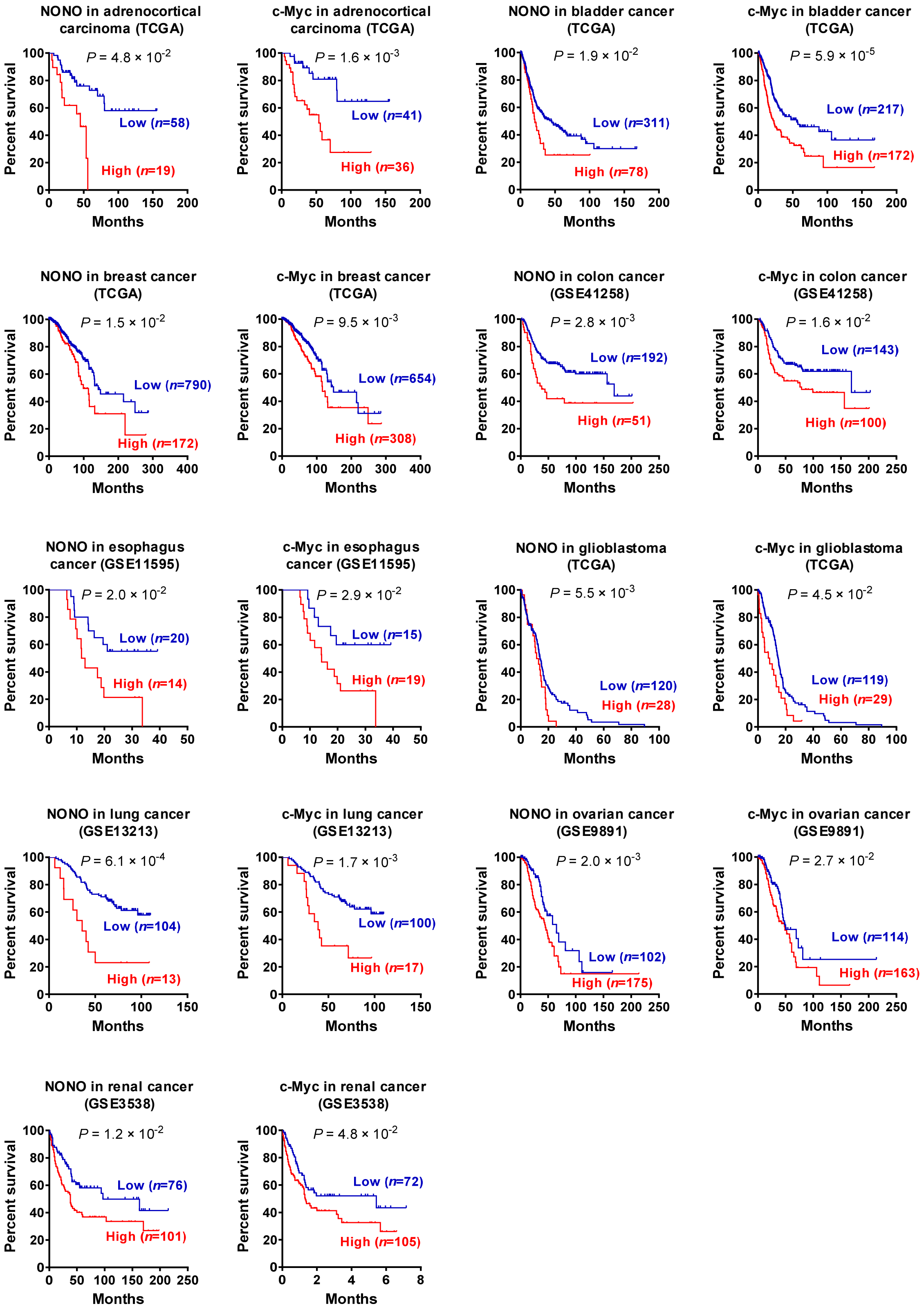

Figure S8. Kaplan-Meier curves of *NONO* and *c-Myc* in multiple cancers. Kaplan-Meier curves indicating the survival of patients with low or high expression of *NONO1* or *c-Myc* in adrenocortical carcinoma, bladder cancer, breast cancer, colon cancer, esophagus cancer, glioblastoma, lung cancer, ovarian cancer, or renal cancer. Log-rank test for survival comparison.

Table S1 Primer sets used for RT-PCR, qPCR, ChIP, and RIP

Primer set	Primers	Sequence	Product size (bp)	Application
circ-hnRNPU (Divergent)	Forward	5'-GTTGTGATGATGATTGGCTTGC-3'	209	RT-PCR
	Reverse	5'-CCACTTGTAGTTAGTGACCAGCC-3'		qPCR, RIP
lin-hnRNPU (Convergent)	Forward	5'-TCCCGCATGTTCTCTGCCACAA -3'	178	RT-PCR
	Reverse	5'-CCAATCATCATCACAACTTCAC-3'		
hsa_circ_0017270 (Divergent)	Forward	5'-TCGGCTATCCATACCCTCGTG-3'	269	RT-PCR
	Reverse	5'-GCTCCAAGAGTTTTTGGGCTTC-3'		
hsa_circ_0017273 (Divergent)	Forward	5'-CGGTGGAGGAAGTGGTGGAAT-3'	235	RT-PCR
	Reverse	5'-CTTCTTTCGGGCAGCAATCTC-3'		
hsa_circ_0017274 (Divergent)	Forward	5'-TGCTGCTGCCCAGAGGAGAAA-3'	261	RT-PCR
	Reverse	5'-ACAGTGGCCGTCCAGCAAGAA-3'		
hsa_circ_0017276 (Divergent)	Forward	5'-TGGTCAGAAGGAAAAGCCATAT-3'	222	RT-PCR
	Reverse	5'-TCACCAAGTAACATTCCACTTG-3'		
β-actin (Divergent)	Forward	5'-AAATCGTGCGTGACATTAAGGAGA-3'	-	RT-PCR
	Reverse	5'-CATACCCCTCGTAGATGGGCA-3'		
β-actin (Convergent)	Forward	5'-TGCCCATCTACGAGGGGTATG-3'	156	RT-PCR
	Reverse	5'-TCTCCTTAATGTCACGCACGATTT-3'		qPCR
GAPDH	Forward	5'-AGAAGGCTGGGGCTCATTTG-3'	258	qPCR
	Reverse	5'-AGGGGCCATCCACAGTCTTC-3'		
U1	Forward	5'-ACTTACCTGGCAGGGGAGATACC-3'	137	qPCR
	Reverse	5'-CCACTACCACAAATTATGCAGTCG-3'		
NONO	Forward	5'-GCAGGAGATTCGGATGGGTC-3'	166	qPCR
	Reverse	5'-GCGTTCAGTTGTTGGTGGGG-3'		
hnRNPU	Forward	5'-GAGGTAAGAAGAAGGCGGAAGG-3'	196	qPCR, RIP
	Reverse	5'-ACAGGTGGCTGAGGAGATTTGG-3'		
GALNT6	Forward	5'-GGATGTGAGCAGCAGAGAGG-3'	200	qPCR
	Reverse	5'-GGGTATAGAACCCAGGGAGG-3'		
FN1	Forward	5'-TGCCAACCTTTACAGACCTAT-3'	171	qPCR
	Reverse	5'-CTTCCAACGGCCTACAGAAT-3'		
MGAT1	Forward	5'-GCTCTACCGCACCGACTTTT-3'	198	qPCR
	Reverse	5'-TGCCCGTGGCTCACACCCTT-3'		
GLUT1	Forward	5'-GATGCGGGAGAAGAAGGTCA-3'	143	qPCR
	Reverse	5'-CGAAGATGCTCGTGGAGTAATAGA-3'		
c-Myc	Forward	5'-TGGTCTTCCCCTACCCTCTCA-3'	205	qPCR
	Reverse	5'-TCTTCCTCATCTTCTTGTTCC-3'		
GALNT2	Forward	5'-AGTCGCCCCTATAAAAACCC-3'	114	qPCR
	Reverse	5'-TTCTCTCCTCCCCACACATC-3'		
FUT2 (-212/-41)	Forward	5'-CGGATGGGGGATGCGACCTT-3'	172	ChIP
	Reverse	5'-GTGTGGCTGGGCTGCGGGAG-3'		
GALNT2 (-640/-422)	Forward	5'-AAACTGCAGCAAGCAGCAGC-3'	219	ChIP
	Reverse	5'-GCACACGTGGGCAAAGAGCC-3'		
GALNT6 (-318/-96)	Forward	5'-TCAGGTGATTCTGACTGGCG-3'	223	ChIP
	Reverse	5'-GGTTTGCTTCTCATTTGTGC-3'		
MGAT1 (-1426/-1286)	Forward	5'-AAGTCTGGCCGGGAGGAGGA-3'	141	ChIP
	Reverse	5'-GAGCTGGGCGAGGGAGTGGG-3'		
ST6GAL1 (-624/-417)	Forward	5'-TGCCTGGACTATGTGTTGGT-3'	208	ChIP
	Reverse	5'-GCCTCATGGGAGGGTTGTAA-3'		
ST6GALNAC5 (-321/-89)	Forward	5'-TTTGTTTTTATCAACATCGC-3'	233	ChIP
	Reverse	5'-AAGACCTCTACATCCCCTTC-3'		
GALNT2	Forward	5'-ACACGCTAGTATTGGCTTCAT-3'	245	RIP
	Reverse	5'-GACCCTTCCTAATTCTTTCCC-3'		
GALNT6	Forward	5'-CCCGGAAAGAAGAGAATTGGA-3'	187	RIP
	Reverse	5'-ATGTTTGGGGACATGAGACAG-3'		

circ-hnRNPU, circular RNA derived from hnRNPU; GAPDH, glyceraldehyde 3-phosphate dehydrogenase; U1, U1 small nuclear 1; NONO, non-POU domain containing octamer binding; hnPNPU, heterogeneous nuclear ribonucleoprotein U; GALNT6, polypeptide N-acetylgalactosaminyltransferase 6; FN1, fibronectin 1; MGAT1, alpha-1,3-mannosyl-glycoprotein 2-beta-N-acetylgluco saminyltransferase; GLUT1, glucose transporter 1; FUT2, fucosyltransferase 2; GALNT2, polypeptide N-acetylgalactosaminyltransferase 2; ST6GAL1, ST6 beta-galactoside alpha-2,6-sialyltransferase 1; ST6GALNAC5, ST6 N-acetylgalactosaminide alpha-2,6-sialyltransferase 5; RT-PCR, reverse transcription PCR; qPCR, quantitative PCR; ChIP, chromatin Immunoprecipitation; RIP, RNA immunoprecipitation.

Table S2 Oligonucleotide sets used for short hairpin RNAs, probe, or guide DNA

Oligo Set	Sequences
sh-Scb	5'-CCGGGCGAACGATCGAGTAAACGGACTCGAGTCCGTTTACTCGATCGTTCGCTTTTT-3' (sense);
	5'-AATTCAAAAAGCGAACGATCGAGTAAACGGACTCGAGTCCGTTTACTCGATCGTTCGC-3' (antisense)
sh-circ-hnRNPU #1	5’-CCGGTAAGATGATGGTTACAGAGAAGCTCGAGCTTCTCTGTAACCATCATCTTTTTTTG-3’ (sense);
	5’-GATCCAAAAAAAGATGATGGTTACAGAGAAGCTCGAGCTTCTCTGTAACCATCATCTTA-3’ (antisense)
sh-circ-hnRNPU#2	5’-CCGGTGGATAAGATGATGGTTACAGACTCGAGTCTGTAACCATCATCTTATCCTTTTTG-3’ (sense);
	5’-GATCCAAAAAGGATAAGATGATGGTTACAGACTCGAGTCTGTAACCATCATCTTATCCA-3’ (antisense)
sh-NONO #1	5'-CCGGGCTGCTACAATGGAAGGAATTCTCGAGAATTCCTTCCATTGTAGCAGCTTTTTG-3' (sense);
	5'-AATTCAAAAAGCTGCTACAATGGAAGGAATTCTCGAGAATTCCTTCCATTGTAGCAGC-3' (antisense)
sh-NONO #2	5'-CCGGGCAGGCGAAGTCTTCATTCATCTCGAGATGAATGAAGACTTCGCCTGCTTTTTG-3'(sense);
	5'-AATTCAAAAAGCAGGCGAAGTCTTCATTCATCTCGAGATGAATGAAGACTTCGCCTGC-3' (antisense)
sh-c-Myc #1	5'-CCGGCCCAAGGTAGTTATCCTTAAACTCGAGTTTAAGGATAACTACCTTGGGTTTTTG-3' (Sense)
	5'-AATTCAAAAACCCAAGGTAGTTATCCTTAAACTCGAGTTTAAGGATAACTACCTTGGG-3' (Antisense)
sh-c-Myc #2	5'-CCGGCAGTTGAAACACAAACTTGAACTCGAGTTCAAGTTTGTGTTTCAACTGTTTTTG-3' (Sense)
	5'-AATTCAAAAACAGTTGAAACACAAACTTGAACTCGAGTTCAAGTTTGTGTTTCAACTG-3' (Antisense)
circ-hnRNPU probe	5’-GGATCTTCTCTGTAACCATCATCTTATCCA-3’ (antisense);
	5’-TGGATAAGATGATGGTTACAGAGAAGATCC-3’ (sense)
circ-hnRNPU guide	5'-CTGGGATCTTCTCTGCATCTTATCCATAAT-3'

hnPNPU, heterogeneous nuclear ribonucleoprotein U; NONO, non-POU domain containing octamer binding.

Table S3 Oligonucleotide sets used for constructs

Oligo Set	Sequences
pLCDH-circ-hnRNPU	5'-CGGAATTCTGAAATATGCTATCTTACAGGTTACAGAGAAGATCCCAGTAAG-3' (sense);<br>5'-CGCGGATCCTCAAGAAAAAATATATTCACCATCATCTTATCCATAATAGTAT-3' (antisense)
pLCDH-lin-hnRNPU	5'-CGGAATTCTGAAATATGCTATCTTACAGGTTACAGAGAAGATCCCAGTAAG-3' (sense);<br>5'-CGCGGATCCGGCATCATCTTATCCATAATAGTAT-3' (antisense)
pCMV-3Tag-1A-NONO	5'-CGCGGATCCATGCAGAGTAATAAAACTTTTAACTTGGAG-3' (sense);<br>5'-GCCGCTCGAGTTAGTATCGGCGACGTTTGTTTGG-3' (antisense)
pCMV-3Tag-1A-NONO (ΔN)	5'-CGCGGATCCAGCCGTCTTTTTGTGGGAAATCTT-3' (sense);<br>5'-GCCGCTCGAGTTAGTATCGGCGACGTTTGTTTGG-3' (antisense)
pCMV-3Tag-1A-NONO (ΔC)	5'-CGCGGATCCATGCAGAGTAATAAAACTTTTAACTTGGAG-3'(sense);<br>5'-GCCGCTCGAGGGTTCCCTTGAATCCTTCCTGCTG-3' (antisense)
pCMV-3Tag-1A-NONO (DBHS)	5'-CGCGGATCCAGCCGTCTTTTTGTGGGAAATCTT-3' (sense);<br>5'-GCCGCTCGAGGGTTCCCTTGAATCCTTCCTGCTG-3' (antisense)
pCMV-3Tag-1A-NONO (DBHS ΔRRM1)	5'-CGCGGATCCGCATCCCTTACAGTTCGAAACCTT-3' (sense);<br>5'-GCCGCTCGAGGGTTCCCTTGAATCCTTCCTGCTG-3' (antisense)
pCMV-3Tag-1A-NONO (DBHS ΔRRM1+2)	5'-CGCGGATCCTTAGATGATGAAGAGGGACTTCCA-3' (sense);<br>5'-GCCGCTCGAGGGTTCCCTTGAATCCTTCCTGCTG-3' (antisense)
pCMV-3Tag-1A-NONO (Coiled-Coil)	5'-CGCGGATCCGCCATGCGCTGGAAGGCACTCATT-3' (sense);<br>5'-GCCGCTCGAGGGTTCCCTTGAATCCTTCCTGCTG-3' (antisense)
pCMV-3Tag-1A-NONO (mNLS)	5'-CCCAAACAAATGTCACCGATACTAACTCGAGGGGGGGCCCGGTAC-3' (sense);<br>5'-TTAGTATCGGTGACATTTGTTTGGGGCAAATTCAGCTCCAGGAGC-3' (antisense)
pGEX-6P-1-NONO	5'-CGCGGATCCATGCAGAGTAATAAAACTTTTAACTTGGAG-3' (sense);<br>5'-GCCGCTCGAGTTAGTATCGGCGACGTTTGTTTGG-3' (antisense)
pGEX-6P-1-NONO (ΔN)	5'-CGCGGATCCAGCCGTCTTTTTGTGGGAAATCTT-3' (sense);<br>5'-GCCGCTCGAGTTAGTATCGGCGACGTTTGTTTGG-3' (antisense)
pGEX-6P-1-NONO (ΔC)	5'-CGCGGATCCATGCAGAGTAATAAAACTTTTAACTTGGAG-3' (sense);<br>5'-GCCGCTCGAGGGTTCCCTTGAATCCTTCCTGCTG-3' (antisense)
pGEX-6P-1-NONO (DBHS)	5'-CGCGGATCCAGCCGTCTTTTTGTGGGAAATCTT-3' (sense);<br>5'-GCCGCTCGAGGGTTCCCTTGAATCCTTCCTGCTG-3' (antisense)
pGEX-6P-1-NONO (DBHS ΔRRM1)	5'-CGCGGATCCGCATCCCTTACAGTTCGAAACCTT-3' (sense);<br>5'-GCCGCTCGAGGGTTCCCTTGAATCCTTCCTGCTG-3' (antisense)
pGEX-6P-1-NONO (DBHS ΔRRM1+2)	5'-CGCGGATCCTTAGATGATGAAGAGGGACTTCCA-3' (sense);<br>5'-GCCGCTCGAGGGTTCCCTTGAATCCTTCCTGCTG-3' (antisense)
pGEX-6P-1-NONO (Coiled-Coil)	5'-CGCGGATCCGCCATGCGCTGGAAGGCACTCATT-3' (sense);<br>5'-GCCGCTCGAGGGTTCCCTTGAATCCTTCCTGCTG-3' (antisense)
pGL3-c-Myc luc	5'-CCCACGTGCATAGCACGTGCTATGCACGTGGATACCACGTGGA-3' (sense);<br>5'-CATGGGGTGCACGTATCGTGCACGATACGTGCACCTATGGTGCACCTTCGA-3' (antisense)
pGL3-ERG luc	5’-CACAGGAAGTGGTAGGAGGAAGTGGTAAGAGGAAGTGGTAGCAGGAAGTGGA-3’ (sense);<br>5’-AGCTTCCACTTCCTGCTACCACTTCCTCTTACCACTTCCTCCTACCACTTCCTGTGGTAC-3’ (antisense)
pGL3-GATA2 luc	5’-CAGAGATAAGAACTGATAACAAAAGATAGGAAGTGATAGCAA-3’ (sense);<br>5’-AGCTTTGCTATCACTTCCTATCTTTTGTTATCAGTTCTTATCTCTGGTAC-3’ (antisense)
pGL3-HNF4A luc	5’-CATGAACTTTGACCTACTGAACTTTGAACTAAGTCCAAAGTCCATAGGTCCAAAGTCCAA-3’ (sense);<br>5’-AGCTTTGGACTTTGGACCTATGGACTTTGGACTTAGTTCAAAGTTCAGTAGGTCAAAGTTCATGGTAC-3’ (antisense)
pGL3-POU5F1 luc	5’-CTTGCAAATGCAATATGATCTGCAAATGGAACGTGATCTGGATATGCATCATAA-3’ (sense);<br>5’-AGCTTTATGATGCATATCCAGATCACGTTCCATTTGCAGATCATATTGCATTTGCAAGGTAC-3’ (antisense)
pGL3-GALNT6 promoter	5'-CGGGGTACCATTTTTAGTAGAGACGGGGT-3' (sense);<br>5'-GCCGCTCGAGGAGGACAGGCATTAGCAGAG-3' (antisense)
pGL3-MGAT1 promoter	5'-CGGGGTACCGCTTTGGCGGAAGCACCATG-3' (sense);<br>5'-GCCGCTCGAGTAGTCCTCGGCTACCCACCCTCAC-3' (antisense)
psiCHECK-2-NONO reporter	5'-TCGAGAGGGATATCTCAGGGACATCTTAGGGATATCTCAGGGAGC-3' (sense);<br>5'-GGCCGCTCCCTGAGATATCCCTAAGATGTCCCTGAGATATCCCTC-3' (antisense)

hnPNPU, heterogeneous nuclear ribonucleoprotein U; NONO, non-POU domain containing octamer binding; DBHS, Drosophila behaviour/human splicing; NLS, nuclear localization signal; ERG, ETS-related gene; GATA2, GATA binding protein 2; HNF4A, hepatocyte nuclear factor 4 alpha; POU5F1, POU class 5 homeobox 1; GALNT6, polypeptide N-acetylgalactosaminyltransferase 6; MGAT1, alpha-1,3-mannosyl-glycoprotein 2-beta-N-acetylgluco saminyltransferase.

Table S4 Mass spectrometry analysis of proteins pulled down by *circ-hnRNPU*

AAMP	CLTC	GRWD1	PNN	TAB2
ABLIM1	CNN2	GTF2F2	PNPT1	TAF9
ACAA1	COPB2	HIRIP3	POP7	TCEA1
ACAA2	COPG1	HIST1H1E	PPIE	TGM2
ACAD9	CORO1A	HIST1H2AC	PPIF	THOC1
ACTC1	CORO1B	HIST1H2BN	PPP1CB	TKT
ACTL6A	CORO1C	HK1	PPP1R10	TLN1
ACTN1	CRIP2	HK2	PPP1R18	TMEM263
ACTR2	CS	HRNR	PPP1R9A	TNKS1BP1
ACTR3	CSRP1	HSDL2	PPP1R9B	TNRC6B
AK1	CSTF1	IFI16	PPP2R1A	TOR1AIP1
AKAP2	CSTF3	INTS7	PRDX2	TOX4
ANLN	CTTN	KCTD3	PRKCSH	TPD52
ANP32B	CTTNBP2NL	KDM1A	PRKRA	TPM4
AP2B1	DBNL	KIAA1211	PRMT1	TPR
APEX1	DDX3X	KIAA1671	PRPF3	TPRN
ARF1	DDX42	KIFC1	PRPF31	TUBB4B
ARFIP1	DDX6	LAMTOR5	PRPF40A	TUBG2
ARL6IP4	DKFZp686J1372	LASP1	PRRC1	TXNL1
ARPC1B	DRG1	LCP1	PSMD9	UBL4A
ARPC2	DYNLT1	LMNB2	RAD51AP1	UPP1
ARPC3	EEF1A1	LMO7	RALY	USP39
ARPC5	EIF2S3	LRRFIP2	RAVER1	WBP2
ARPC5L	EIF3D	LSM4	RBBP4	WDR33
ASNA1	EIF3I	MDH1	RPL8	WDR43
ATP5J	EIF3K	MED1	RPS17	WDR82
ATP6V1A	EIF3S3	MGMT	RPS26	WIZ
ATXN2	EIF4H	MRPS11	RPS7	XRN2
BAIAP2	ELOB	MT2A	RTCB	YWHAB
BTF3	ELOC	MYDGF	SAP30BP	ZFR
C14orf166	EPB41L2	MYH9	SEH1L	ZNF787
C19orf43	EPS15L1	NAT10	SEPHS1	ZNF830
CACYBP	EXOSC10	NCBP3	SH3D19	
CAND1	EXOSC2	NDUFS6	SH3PXD2B	
CAPZA2	EXOSC4	NDUFV1	SHKBP1	
CAPZB	EXOSC6	NOMO1	SIPA1L1	
CAT	EZR	NONO	SLAIN2	
CBLL1	FAM120A	NSF	SLC9A3R2	
CCDC137	FEN1	NUP37	SMARCE1	
CCT7	FHL2	NUP58	SMTN	
CD109	FIP1L1	NXF1	SNRPE	
CD2AP	FLNB	OLA1	SORBS3	
CDC42	FNBP4	OSBPL11	SPOUT1	
CDKN2AIP	FOXK1	PAICS	SPTBN1	
CHAMP1	FOXK2	PALLD	SQOR	
CHCHD2P9	FRG1	PARD3	SRP72	
CIAO1	FYTTD1	PAWR	SS18	
CKAP2	G3BP2	PDIA6	ST13	
CLIC1	GATAD1	PDLIM2	SUGP1	
CLTB	GNB2	PDLIM7	SVIL	
